# Expression of circular RNAs in myelodysplastic neoplasms and their association with mutations in the splicing factor gene *SF3B1*

**DOI:** 10.1002/1878-0261.13486

**Published:** 2023-07-17

**Authors:** Iva Trsova, Andrea Hrustincova, Zdenek Krejcik, David Kundrat, Aleš Holoubek, Karolina Staflova, Lucie Janstova, Sarka Vanikova, Katarina Szikszai, Jiri Klema, Petr Rysavy, Monika Belickova, Monika Kaisrlikova, Jitka Vesela, Jaroslav Cermak, Anna Jonasova, Jiri Dostal, Jan Fric, Jan Musil, Michaela Dostalova Merkerova

**Affiliations:** ^1^ Department of Genomics Institute of Hematology and Blood Transfusion Prague Czech Republic; ^2^ Department of Genetics and Microbiology, Faculty of Science Charles University Prague Czech Republic; ^3^ Department of Proteomics Institute of Hematology and Blood Transfusion Prague Czech Republic; ^4^ Department of Biochemistry Institute of Organic Chemistry and Biochemistry of the Czech Academy of Sciences Prague Czech Republic; ^5^ Department of Modern Immunotherapy Institute of Hematology and Blood Transfusion Prague Czech Republic; ^6^ Department of Cell Biology, Faculty of Science Charles University Prague Czech Republic; ^7^ Department of Immunomonitoring and Flow Cytometry Institute of Hematology and Blood Transfusion Prague Czech Republic; ^8^ Department of Computer Science Czech Technical University Prague Czech Republic; ^9^ Laboratory of Anemias Institute of Hematology and Blood Transfusion Prague Czech Republic; ^10^ First Department of Medicine General University Hospital Prague Czech Republic; ^11^ International Clinical Research Center of St. Anne's University Hospital (FNUSA‐ICRC) Brno Czech Republic

**Keywords:** circular RNA, myelodysplastic neoplasms, SF3B1, splicing, ZEB1

## Abstract

Mutations in the splicing factor 3b subunit 1 (*SF3B1*) gene are frequent in myelodysplastic neoplasms (MDS). Because the splicing process is involved in the production of circular RNAs (circRNAs), we investigated the impact of *SF3B1* mutations on circRNA processing. Using RNA sequencing, we measured circRNA expression in CD34+ bone marrow MDS cells. We defined circRNAs deregulated in a heterogeneous group of MDS patients and described increased circRNA formation in higher‐risk MDS. We showed that the presence of *SF3B1* mutations did not affect the global production of circRNAs; however, deregulation of specific circRNAs was observed. Particularly, we demonstrated that strong upregulation of circRNAs processed from the zinc finger E‐box binding homeobox 1 (*ZEB1*) transcription factor; this upregulation was exclusive to *SF3B1*‐mutated patients and was not observed in those with mutations in other splicing factors or other recurrently mutated genes, or with other clinical variables. Furthermore, we focused on the most upregulated *ZEB1*‐circRNA, hsa_circ_0000228, and, by its knockdown, we demonstrated that its expression is related to mitochondrial activity. Using microRNA analyses, we proposed miR‐1248 as a direct target of hsa_circ_0000228. To conclude, we demonstrated that mutated *SF3B1* leads to deregulation of *ZEB1*‐circRNAs, potentially contributing to the defects in mitochondrial metabolism observed in *SF3B1*‐mutated MDS.

## Graphical abstract

The authors investigated circular RNA (circRNA) processing in patients with myelodysplastic neoplasms and observed upregulation of circRNAs derived from the zinc finger E‐box binding homeobox 1 (*ZEB1*) gene exclusively in patients with mutated splicing factor 3b subunit 1 (*SF3B1*). By knocking down the most upregulated ZEB1‐circRNA, hsa_circ_0000228, and using functional analyses, the authors demonstrated that its expression is related to mitochondrial activity and propose miR‐1248 as a direct target of this circRNA.

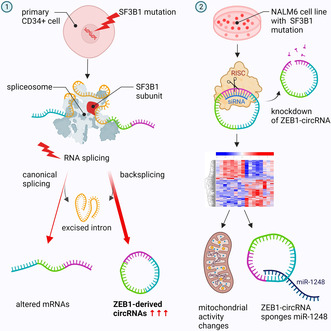

**Abbreviations**
AML‐MRC: acute myeloid leukaemia with myelodysplasia‐related changes
BM: bone marrow
circRNAs: circular RNAs
CPM: counts per million
DEA: differential expression analysis
DNMT: DNA methyltransferase
EB: excess of blasts
ECAR: extracellular acidification rate
EMT: epithelial‐to‐mesenchymal transition
FDR: false discovery rate
GSEA: gene set enrichment analysis
HSC: hematopoietic stem cell
IPSS‐R: revised international prognostic scoring system
MDS: myelodysplastic neoplasms
MLD: multilineage dysplasia
MNC: mononuclear cell
n.a.: not analysed
NCBI: National Center for Biotechnology Information
OCR: oxygen consumption rate
PI: propidium iodide
RS: ring sideroblasts
RT‐qPCR: reverse transcription quantitative polymerase chain reaction
siRNA: small interfering RNA
SLD: single lineage dysplasia
SRA: Sequence Read Archive
VAF: variant allele frequency

## 1 Introduction

Myelodysplastic neoplasms (MDS) represent hematopoietic stem cell (HSC) malignancies characterised by ineffective hematopoiesis, peripheral blood cytopenia and a tendency for leukaemic transformation. Acute myeloid leukaemia with myelodysplasia‐related changes (AML‐MRC) develops in 30–40% of MDS patients. MDS is a heterogeneous disease with several diagnostic subtypes [1], and the patient prognosis can be very different [2].

Significant progress in the study of MDS pathogenesis has occurred in recent years. Numerous breakthrough studies have described the presence of multiple somatic mutations in MDS. These mutations were characterised in several key components of the spliceosome machinery, regulators of DNA methylation, chromatin modification, transcription, signalling and cell cycle control [3]. Mutations in RNA splicing factors (*SF3B1*, *SRSF2*, *U2AF1*, *U2AF2* and *ZRSR2*) represent the most common class of genetic alterations in MDS, as they are present in 50–60% of MDS patients. The splicing factor 3b subunit 1 (*SF3B1*) gene is mutated in approximately 15–28% of all MDS patients and is thus the most frequently mutated gene described in MDS [4]. Regarding the outcome, patients with *SF3B1* mutations showed significantly longer survival and a lower probability of disease progression. Importantly, the phenotypic and clinical specifics of these patients recently led to the proposal of MDS with low blasts and an *SF3B1* mutation (MDS‐*SF3B1*) as a new MDS sybtype [1]. Conversely, mutations in the *SRSF2* gene are likely to predict shorter survival and a higher probability of disease progression [4]. Splicing factor mutations are heterozygous and largely mutually exclusive to each other. In addition to the above‐mentioned splicing factors, mutations in several other genes, such as the epigenetic modifiers *DNMT3A* [5] or *TET2* [6], have been described as related to aberrant splicing in MDS.

Pre‐mRNA splicing, a process of excision of intronic sequences catalysed by a spliceosome complex, can create a range of alternative transcripts by varying the exon composition of the same pre‐mRNA. Analyses of the cancer transcriptome have suggested that cancer cells exhibit ‘noisier; splicing than their normal tissue counterparts [7]. However, splicing not only makes the linear transcriptome more diverse but can also result in RNA circularisation. Circular RNAs (circRNAs) are covalently enclosed RNAs produced during the so‐called ‘backsplicing’ process, which does not follow the canonical 5′–3′ order but creates backsplice junctions (BSJs) [8]. Inhibition of canonical splicing has been proven to reduce circRNA levels, providing evidence for a role for the spliceosome in circRNA biogenesis [9].

Although the functions of most circRNAs remain largely unexplored, they can sequester miRNAs or proteins, modulate RNA polymerase II transcription, interfere with splicing and even be translated to produce polypeptides [10]. The expression of circRNAs does not always correlate with the expression of linear transcripts, indicating that circRNA expression is specifically regulated and that the spliceosome must be able to discriminate between canonical splicing and backsplicing [11]. Many studies have demonstrated important roles of circRNAs in oncogenesis (reviewed by Zhang and Xin [12]). Although RNA splicing has been recognised as one of the key processes for MDS development, little information about the role of circRNAs in MDS is known [13, 14]. Both of the studies published to date have focused on the potential of circRNAs to become biomarkers of MDS progression [13] and predict response to azacitidine therapy [14].

Here, we explored circRNA profiles in a large cohort of MDS patients and studied to what extent aberrant splicing observed in MDS also affects the formation of circRNAs. We showed that circRNAs processed from the zinc finger E‐box binding homeobox 1 (*ZEB1*) transcription factor are specifically upregulated due to *SF3B1* mutations in MDS patients and that deregulation of these circRNAs is associated with mitochondrial processes.

## 2 Materials and methods

### 2.1 Patients

The study included 98 samples of bone marrow (BM) CD34+ cells. The samples were obtained from 13 hematologically healthy donors (control samples) and 85 patients with a diagnosis of MDS or AML‐MRC during routine clinical assessment (from January 2007 to February 2020) at the Institute of Hematology and Blood Transfusion and at the General Faculty Hospital, both in Prague. All the patients included in the study had a known history without any previous malignancy, chemotherapy or radiation therapy; moreover, none of the patients received HSC transplantation. The patients' diagnoses were assessed based on the standard WHO 2016 classification criteria [15], and all the patients were classified according to the revised international prognostic scoring system (IPSS‐R) categories [16] at the time of sample collection. Written informed consent was obtained from each tested subject, and the study was approved by the Institutional Scientific Board and the Local Ethics Committee (licence number EK 2/GA CR/03/2019) and performed in accordance with the ethical standards of the Declaration of Helsinki. The detailed clinical and laboratory characteristics of the cohort, including the patient classification into subgroups, IPSS‐R categories, blood counts, cytogenetics and mutational status, are summarised in Table S1. Because of the purposes of RNA sequencing, only patients with a high variant allele frequency (VAF > 10%) of detected mutations were included in the study cohort. The mean VAF of the *SF3B1* mutation was 37% (range 10–50%).

### 2.2 Cell separation and nucleic acid extraction

Mononuclear cells (MNCs) were separated from BM aspirates by Ficoll‐Paque density gradient centrifugation (GE Healthcare, Munich, Germany). CD34+ cells were isolated from MNCs using a magnetic cell separation system from Miltenyi Biotec (Bergisch Gladbach, Germany). The acid‐guanidine‐phenol‐chloroform method was used to extract total RNA, and the samples were incubated with DNase I (Qiagen, Hilden, Germany) to prevent genomic DNA contamination. RNA was quantified using Qubit 3 Fluorometer (Thermo Fisher Scientific, Waltham, MA, USA), and the RNA integrity was assessed using Agilent 4200 TapeStation (Agilent Technologies, Santa Clara, CA, USA).

### 2.3 Mutational screening

Mutational screening was performed as a part of routine clinical assessment using the TruSight Myeloid Sequencing Panel Kit (Illumina, San Diego, CA, USA) containing 568 amplicons of 54 genes associated with myeloid malignancies as previously described [17, 18].

### 2.4 RNA sequencing

Ribosomal RNA was depleted from total RNA by a RiboCop rRNA Depletion Kit (Lexogen, Vienna, Austria), and libraries were constructed with an NEBNext Ultra II RNA Library Prep Kit (New England Biolabs, Ipswich, MA, USA). Library quality and size were assessed using Agilent 4200 TapeStation and quantified with Qubit 3 Fluorimeter. Libraries were subsequently pooled and 2 × 100 bp paired‐end‐sequenced on a NovaSeq 6000 instrument (Illumina).

fastq files were subjected to initial quality control by fastqc [19]. Adaptor trimming and low‐quality sequence removal were performed by trimmomatic [20]. For general identification of linear gene transcripts, the reads were aligned to the human genome version hg38 using star [21], which considers mRNA splicing. The transcripts were then counted by the stringtie tool [22] and outputted in the form of an expression matrix as counts per million (CPM). For circRNA identification, the reads were mapped to the human genome hg19 using bwa [23] because the genomic coordinates of circRNAs were provided in the circBase database in this version. The mapped data were further processed by ciri2 [24] and ciriquant [25] software. ciri2 software provided us with BSJ counts and junction ratios of specific circRNA, which were then used by CIRIquant to perform differential expression analysis (DEA) and to visualise circRNA splicing events (Sashimi plots) using ularcirc [26].

All other subsequent statistical analyses were performed in the r statistical environment with appropriate bioconductor packages. The pcamethods package was used for sample clustering. DEA for classic gene transcripts was performed using edger. For DEA, we retained only the transcripts that had at least 5 CPM in the number of libraries corresponding at least to half of the smallest sample group. In standard settings, only the transcripts with false discovery rate (FDR) < 0.05 (*P* value adjusted for multiple testing) were considered as significantly deregulated. Graphical output in the form of boxplots and heatmaps was created using the ggplot2 and pheatmap packages in r software. The functional changes related to gene deregulation were assessed using gene set enrichment analysis (GSEA).

### 2.5 Cell cultures and *in vitro* assays

K562 (RRID:CVCL_0004; DSMZ, Leibniz, Germany), wild‐type NALM6 (*SF3B1*wt; RRID:CVCL_0092; Horizon Discovery, Cambridge, UK) and isogenic NALM6 cells with CRISPR/Cas9‐edited *SF3B1* ^K700E^ mutation (*SF3B1*mut; cat. ID: HD 115‐104; Horizon Discovery) were grown in RPMI‐1640 medium supplemented with 10% FBS (both Thermo Fisher Scientific) and incubated at 37 °C in 5% CO_2_. All the cell lines have been authenticated and certified by their suppliers, and all experiments were performed with mycoplasma‐free cells (tested by MycoAlert PLUS Mycoplasma Detection Kit; Lonza, Basel, Switzerland). Small interfering RNAs (siRNAs) against *SF3B1* (Silencer siRNA, ID s23850) and hsa_circ_0000228 (target sequence 5′‐CCTTGTTCTCTAAAG|TTTTCA‐3′), and scrambled‐siRNA (scm‐siRNA; Silencer Select Negative Control, ID 4390843) were purchased from Thermo Fisher Scientific. Transfection of siRNAs was performed by an Amaxa 4D‐Nucleofector device with SF 4D‐Nucleofector™ X Solution (Lonza) using the CV104 program. To compare the stability of linear RNAs and circRNAs, actinomycin D (Sigma‐Aldrich, Steinheim, Germany) was used to block RNA transcription. Cells were treated with actinomycin D at a final concentration of 2.5 μg·mL^−1^ for 48 h, and DMSO was applied as a negative control. For specific cleavage of linear transcripts, RNA samples were incubated for 30 min at 37 °C with or without 1 U of RNase R (Biosearch Technologies, Hoddesdon, UK).

### 2.6 Reverse transcription quantitative PCR

Reverse transcription quantitative PCR (RT‐qPCR) was used to quantify the expression of individual transcripts. SuperScript IV VILO Master Mix was utilised for cDNA synthesis. TaqMan gene expression assays with TaqMan Universal Mastermix II with UNG were applied for quantitative PCR using a StepOnePlus instrument (all Thermo Fisher Scientific). For quantification of linear mRNAs, standard TaqMan gene expression assays were purchased, and for quantification of circRNAs, we designed TaqMan assays specific for BSJs using the Thermo Fisher Custom TaqMan Assay Design Tool. Sequences of these designed primers and probes are listed in Table S2. For the purpose of miRNA quantification, TaqMan microRNA cDNA synthesis kit and standard TaqMan microRNA expression assays (Thermo Fisher Scientific) were used. For normalisation of the raw *C*_t_ data, the *HPRT1* reference gene (linear mRNAs and circRNAs) and *RNU48* (miRNAs) were selected based on our previous testing [27], and the data were further processed by the $2^{-\Delta \Delta C_{t}}$ method.

### 2.7 Western blot

For protein detection, cells were harvested and lysed on ice in CelLytic™ M lysis buffer (Sigma‐Aldrich) supplemented with 25 U·mL^−1^ of benzonase (Millipore, Burlington, MA, USA) and further boiled for 10 min in loading buffer (100 mm Tris–HCl, 4% SDS, 3% 2‐mercaptoethanol, 0.2% bromophenol blue, 20% glycerol, pH 6.8). Cell lysates were loaded on 4–15% precast polyacrylamide gel and transferred to polyvinylidene fluoride (PVDF) membrane (both BioRad, Hercules, CA, USA). The membrane was probed with 1 : 5000 dilution of anti‐ZEB1 rabbit polyclonal antibody (Sigma‐Aldrich; #HPA027524) or 1 : 5000 dilution of anti‐SF3B1 rabbit monoclonal antibody (Invitrogen, Waltham, MA, USA; #MA5‐34649). Equal protein loading and transfer was verified by mouse monoclonal anti‐GAPDH antibody (Invitrogen; #MA5‐15738) on the same membrane. As secondary antibodies conjugated with horseradish peroxidase, we used goat anti‐mouse (Sigma‐Aldrich; #A4416) and goat anti‐rabbit (Sigma‐Aldrich; #AP307P) antibodies. For visualisation, we used a SuperSignal™ West Femto Maximum Sensitivity Substrate (Thermo Fisher Scientific) and LAS‐3000 imager (Fujifilm, Minato, Japan). The signal intensity was quantified using the imagequant tl software (Cytiva, Marlborough, MA, USA).

### 2.8 Seahorse analysis

Glycolysis and mitochondrial respiration rates were measured on Seahorse XFp Analyzer using a Seahorse XFp Glycolysis Stress Test Kit and a Seahorse XFp Cell Mito Stress Test Kit (all Agilent Technologies). NALM6 cells were seeded at a density of 180 000 cells per well in Agilent XFp PDL miniplates in XF RPMI Base Medium pH 7.4 and supplemented with 2 mm l‐glutamine for the Glycolysis Stress Test or 1 mm pyruvate, 10 mm glucose and 2 mm l‐glutamine for the Mito Stress Test (all Agilent Technologies). The final concentrations of the injected drugs were 10 mm glucose, 1 μm oligomycin, and 50 mm 2‐deoxyglucose or 1 μm oligomycin, 0.5 μm FCCP, and 0.5 μm rotenone + antimycin A and 50 mm 2‐DG mix for the Glycolysis Stress Test and Mito Stress Test, respectively (all Agilent Technologies).

### 2.9 Cell viability, cell cycle and proliferation

Ordinary cell count and viability data were assessed using Trypan Blue exclusion assay (Thermo Fisher Scientific). Cell viability related to metabolic activity was further evaluated using the AlamarBlue assay (Life Technologies, Grand Island, NY, USA), which measures the reducing power of living cells, following the manufacturer's instructions. Briefly, 50 μL of suspension of 1 × 10^5^ NALM6 cells was incubated with AlamarBlue reagent (5 μL) at 37 °C for 2 h. Fluorescence intensity was measured on microplate reader BMG FLUOstar Galaxy (MTX Lab Systems, Vienna, VA, USA). Cell cycle analysis was performed using propidium iodide (PI). Briefly, cells were fixed with 70% ice‐cold ethanol for 2 h, washed and stained with a mixture of 10 μg PI (Sigma‐Aldrich) and 157 μg RNase A (Qiagen) in 200 μL of PBS for 30 min. PI staining was measured on a BD FACSymphony A5 flow cytometer (BD Biosciences, Franklin, NJ, USA) using 561 nm excitation and YG 610/20 detection channel and analysed by flowjo 10.8.1. software (BD Biosciences). Proliferation assay was performed using CellTrace™ Violet (Thermo Fisher Scientific, Invitrogen, Waltham, MA, USA) following the manufacturer's instructions. Briefly, NALM6 cells were harvested and 1 × 10^6^ cells·mL^−1^ was stained with 0.5 μm·mL^−1^ of CellTrace™ Violet for 20 min. Stained cells (1 × 10^6^ cells·mL^−1^) were plated in RPMI‐1640 medium supplemented with 10% FBS. One‐third of the cell suspension was harvested at time points 0, 48 and 72 h and stained with LIVE/DEAD™ Fixable Blue Dead Cell Stain Kit (Thermo Fisher Scientific, Invitrogen). Spectral cytometer Cytek® Aurora (Cytek Biosciences, Fremont, CA, USA) was used for cell acquisition, and flowjo 10.8.0 was used for data analysis.

### 2.10 Luciferase reporter assay

To confirm the interaction between hsa_circ_0000228 and miR‐1248, we performed luciferase reporter assay with pmirGLO Dual‐Luciferase miRNA Target Expression Vector (Promega, Madison, Wisconsin, USA). For cloning, two pairs of sense and antisense DNA oligonucleotides containing the predicted miR‐1248 target sequence (5′AAACTAGCGGCCGCTTCTTACAGGATTTCAAGAAGGAAAGCCT3′, 3′TTTGATCGCCGGCGAAGAATGTCCTAAAGTTCTTCCTTTCGGAGATC5′; target sequence underlined) and a mismatch sequence (5′AAACTAGCGGCCGCTTCTTACAGGATTTCATTAGGAAAGCCT3′, 3′TTTGATCGCCGGCGAAGAATGTCCTAAAGTAATCCTTTCGGAGATC5′) as a negative control were designed and ordered (Thermo Fisher Scientific). The oligonucleotides were annealed and ligated into the pmirGLO vector following the manufacturer's instructions. Successful insertion of the fragments was validated by enzyme digestion and Sanger sequencing. K562 cell line was cotransfected with 300 ng pmirGLO vector with the insert and 200 nm miR‐1248 (Thermo Fisher Scientific) using Amaxa 4D Nucleofector. Multiple negative controls, including mismatch insert vector, empty vector and scrambled miRNA control (Thermo Fisher Scientific), were tested. Six hours after nucleofection, luciferase assay using Dual‐Glo® Luciferase Assay System (Promega) was performed following the manufacturer's instructions. Firefly and Renilla luciferase luminiscence was measured by Tecan Spark microplate reader (Life Sciences, St. Petersburg, FL, USA). For data normalisation, Firefly luciferase signal intensity was related to Renilla luciferase luminescence.

### 2.11 Identification of miR‐1248 targets

MicroRNA target identification was performed for miR‐1248 downloading validated target gene list from miRTarBase Release 9.0 [28]. DAVID tool was utilised for Kyoto Encyclopedia of Genes and Genomes (KEGG) pathway enrichment analysis of the miR‐1248 target gene list [29].

### 2.12 Statistical analyses

Statistical analyses were performed using graphpad prism v9 software (GraphPad Software Inc., San Diego, CA, USA). Student's *t*‐test was used to compare continuous variables between different groups of samples. Correlation analysis was performed by computing Pearson's correlation coefficients (*r*). Differences were considered statistically significant at *P* < 0.05.

## 3 Results

### 3.1 circRNA expression is deregulated in MDS cells

We performed high‐depth RNA sequencing of 98 rRNA‐depleted samples obtained from CD34+ BM cells isolated from 78 MDS patients, 7 AML‐MRC patients and 13 healthy controls. On average, we detected 77 M of raw reads per sample, 87% of which uniquely mapped to the human hg38 genome. Raw data were deposited in the National Center for Biotechnology Information (NCBI) Sequence Read Archive (SRA) database (BioProject ID PRJNA896500). After data normalisation and filtration, we detected the expression of 16 181 genes and 41 747 transcript variants (on average, 2.6 transcript variants were detected per gene). For circRNA analysis, we realigned the data to the hg19 genome and mapped the sequences to the circBase database. On average, 3.7% of reads per sample mapped to circRNAs, and after data normalisation and filtration, we identified 8620 circRNAs.

After processing raw data, we performed three different DEAs, in which we compared the expression data of: (a) patient samples and the samples of healthy controls, (b) samples from higher‐ and lower‐risk MDS patients, and (c) samples of patients with an *SF3B1* mutation and those with no mutation detected. Table S3 shows the numbers of differentially represented features (genes, individual transcripts and circRNAs) in all three DEAs. As expected, the lowest numbers of differentially expressed features were found in the case of circRNAs because their input number was also minor (compared to genes and transcripts). Furthermore, patients with mutated *SF3B1* (i.e., the results of the third DEA in comparison to the results of the first and second DEAs) showed lower numbers of differentially expressed features (420 deregulated genes, 1060 transcript variants and 40 circRNAs, raw *P* < 0.01), which may relate to more subtle phenotypic differences that are associated with mutational status.

**Fig. 1:**
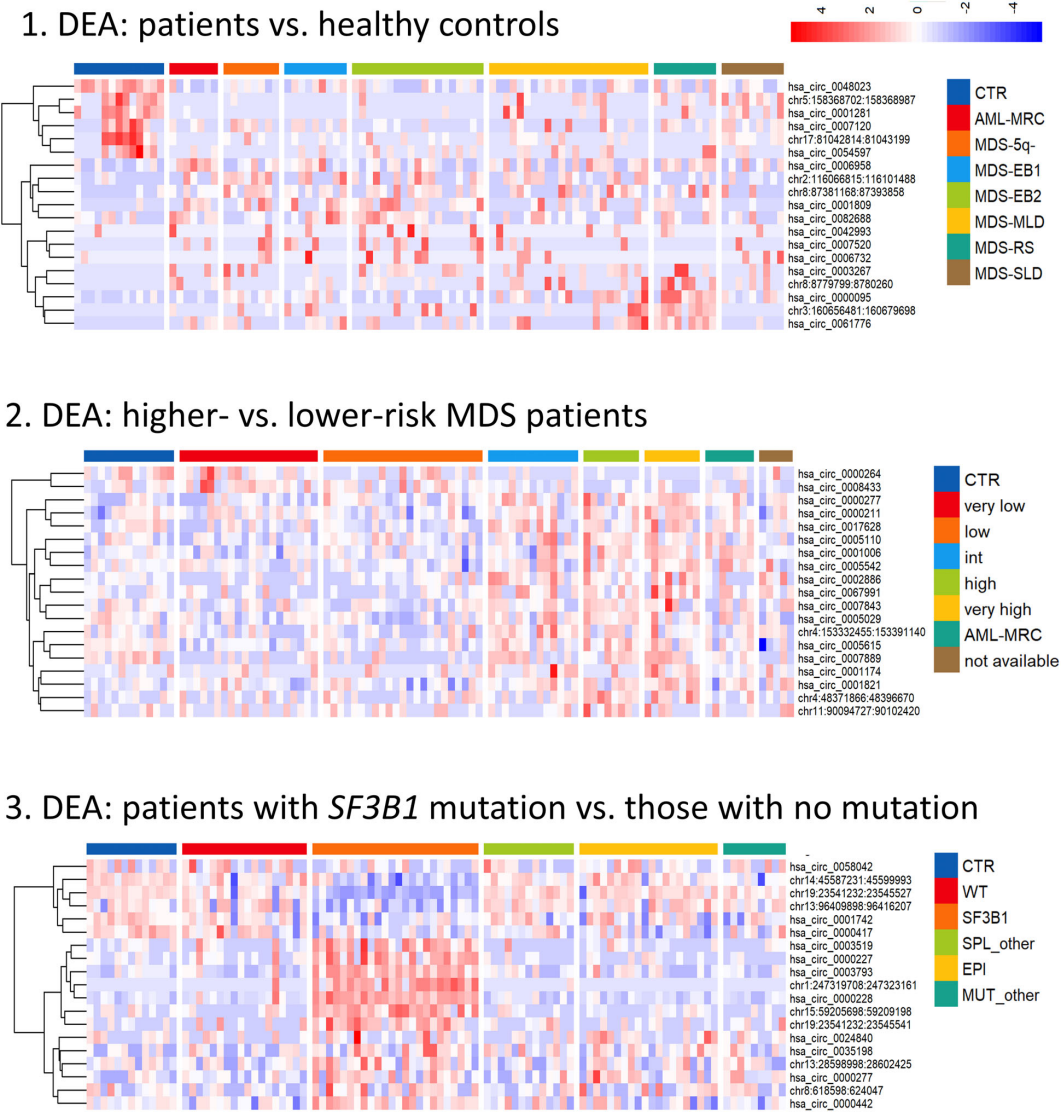
Twenty most significantly deregulated circRNAs in differential expression analyses (DEA) between: (1) patients and healthy controls, (2) higher‐ and lower‐risk MDS patients and (3) patients with an *SF3B1* mutation and those with no mutation detected. AML‐MRC, acute myeloid leukaemia with myelodysplasia‐related changes; CTR, healthy control; EPI, mutations in epigenetic factor genes; MDS‐5q‐, MDS with del(5q); MDS‐EB I/II, MDS with excess of blasts type I/II; MDS‐MLD, MDS with multilineage dysplasia; MDS‐RS, MDS with ring sideroblasts; MDS‐SLD, MDS with single lineage dysplasia; MUT_other, any other mutations; SPL_other, mutations in splicing factor genes other than *SF3B1*; WT, wild‐type.

Our major focus was further placed on the circRNAs with differential expression in specific groups of MDS patients. The majority of these circRNAs were of exonic origin and were produced from protein‐coding genes. Interestingly, we observed strong trend of circRNA overexpression in higher‐risk patients compared to healthy controls and lower‐risk patients. The most significantly deregulated circRNAs in each of the three DEA analyses are listed in Table 1, and their heatmaps are plotted in Fig. 1.

**Table 1:** Ten most significantly deregulated circRNAs in various DEA settings. (A) Patients and healthy controls. (B) Higher‐ and lower‐risk MDS patients. (C) Patients with an *SF3B1* mutation and those with no mutation detected. The top 10 circRNAs were selected and sorted according to FDR values.

Coordinates	circRNA_ID	Gene_name	Circ_type	logFC	*P* value	FDR
**(A) Patients vs. healthy controls**					
chr17:81042814|81043199	–	METRNL	Exon	−3.39	8.93E‐19	9.58E‐15
chr5:158368702|158368987	–	EBF1	Intron	−2.08	1.08E‐05	5.81E‐02
chr3:160656481|160679698	–	PPM1L	Intron	3.22	5.85E‐05	2.06E‐01
chr7:102398301|102401858	hsa_circ_0007520	FAM185A	Exon	3.14	9.12E‐05	2.06E‐01
chr3:35721279|35732497	hsa_circ_0001281	ARPP21	Exon	−1.89	9.97E‐05	2.06E‐01
chr8:70667659|70674110	hsa_circ_0001809	SLCO5A1	Exon	2.72	1.25E‐04	2.06E‐01
chr1:2234417|2236024	hsa_circ_0007120	SKI	Exon	−1.78	1.35E‐04	2.06E‐01
chr2:116066815|116101488	–	DPP10	Exon	2.25	1.80E‐04	2.11E‐01
chr8:87381168|87393858	–	WWP1	Exon	2.65	1.93E‐04	2.11E‐01
chr7:139746684|139757834	hsa_circ_0082688	PARP12	Exon	2.81	2.21E‐04	2.11E‐01
**(B) Higher‐ vs. lower‐risk MDS**					
chr10:125798031|125806240	hsa_circ_0000264	CHST15	Exon	−2.56	1.29E‐07	6.31E‐04
chr12:83250789|83251359	hsa_circ_0002886	TMTC2	Exon	1.85	1.40E‐06	3.43E‐03
chr11:14793483|14810788	hsa_circ_0000277	PDE3B	Exon	1.49	3.16E‐06	4.47E‐03
chr1:86241357|86252144	hsa_circ_0005110	COL24A1	Exon	1.57	3.65E‐06	4.47E‐03
chr10:7318854|7327916	hsa_circ_0000211	SFMBT2	Exon	1.14	6.38E‐06	6.26E‐03
chr2:55209651|55214834	hsa_circ_0001006	RTN4	Exon	1.12	1.04E‐05	8.51E‐03
chr20:58755108|58755971	hsa_circ_0001174	MIR646HG	Intron	1.75	1.42E‐05	9.96E‐03
chr4:48371866|48396670	–	SLAIN2	Exon	1.64	1.69E‐05	1.01E‐02
chr3:171969050|172025291	hsa_circ_0067991	FNDC3B	Exon	1.71	1.86E‐05	1.01E‐02
chr16:68155890|68157024	hsa_circ_0005615	NFATC3	Exon	1.01	2.37E‐05	1.16E‐02
**(C) SF3B1 mutation vs. no mutation**					
chr19:23541232|23545527	–	ZNF91	Exon	−2.51	2.48E‐11	8.47E‐08
chr10:31661947|31676727	hsa_circ_0003793	ZEB1	Exon	2.49	1.91E‐08	3.26E‐05
chr1:247319708|247323161	–	ZNF124	Exon	3.05	6.59E‐08	7.51E‐05
chr13:96409898|96416207	–	DNAJC3	Exon	−2.20	1.04E‐06	8.87E‐04
chr10:31644076|31676195	hsa_circ_0003519	ZEB1	Exon	2.35	5.46E‐05	3.73E‐02
chr15:59205698|59209198	–	SLTM	Exon	2.12	1.34E‐04	6.68E‐02
chr2:215609791|215646233	hsa_circ_0058042	BARD1	Exon	−2.33	1.37E‐04	6.68E‐02
chr8:618598|624047	–	ERICH1	Exon	1.84	1.86E‐04	7.95E‐02
chr11:128932175|128936763	hsa_circ_0024840	ARHGAP32	Exon	2.01	2.69E‐04	1.02E‐01
chr15:50330965|50366382	hsa_circ_0035198	ATP8B4	Exon	1.57	3.15E‐04	1.05E‐01

### 3.2 *SF3B1* mutations alter linear splicing/backsplicing of particular genes

To study alterations in backsplicing in relation to mutated *SF3B1*, we further focused on expression changes between patients with an *SF3B1* mutation and those with no mutation detected. We plotted a Venn diagram of differentially expressed genes, transcript variants and circRNAs (raw *P* < 0.05) and searched for specificities in transcript variants within individual gene loci (Fig. S1). Although there were intersections among the three groups, the majority of the features were uniquely deregulated in only one of the features studied. The most interesting set of gene loci consisted of those whose expression was not changed at the whole gene level but showed alternative splicing, particularly regarding backsplicing, suggesting that processing of these circRNAs was specifically regulated regardless of the expression of their host genes. The list of circRNAs whose levels changed uniquely without affecting the expression of linear transcripts of host genes included *ATM*, *CBL*, *ERCC5*, *ETV6*, *FLT3* and *MAPK6*. Furthermore, gene loci that changed the production of both alternative transcripts and circRNAs along with intact expression at the whole gene level included *CDK14*, *KDM1A* and *ZEB1*. Table S4 provides a full list of these transcripts.

### 3.3 *ZEB1*‐circRNAs are upregulated in *SF3B1*‐mutated MDS

**Fig. 2:**
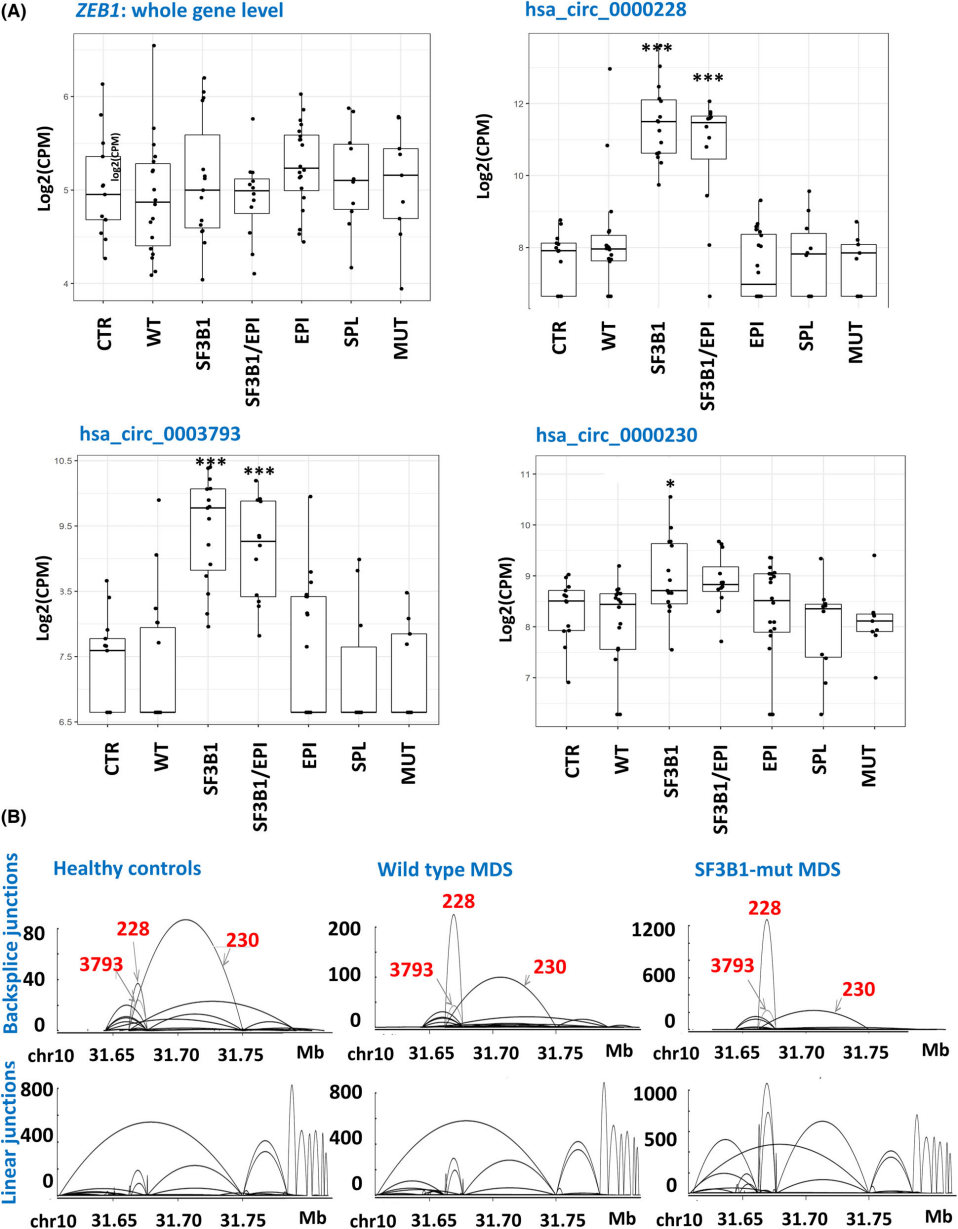
Expression of *ZEB1* analysed by RNA sequencing. (A) Expression of linear *ZEB1* transcript (CPM of all RNA sequencing reads mapping to this gene) and those of three selected *ZEB1*‐processed circRNAs (CPM of reads mapping to specific circRNA junctions) in patient groups stratified based on their mutational status. Data are shown as the mean ± SD. (B) Variants and read numbers of splice junctions detected in *ZEB1* transcripts (top – backsplice junctions, bottom – linear junctions) in a representant healthy control, a wild‐type patient and a patient with an *SF3B1* mutation. The arches specify junction boundaries detected within the transcripts. 228, hsa_circ_0000228; 230, hsa_circ_0000230; 3793, hsa_circ_0003793; CPM, counts per million; CTR, healthy control; EPI, mutations in epigenetic factor genes; MUT, any other mutations; SF3B1/EPI, comutations in *SF3B1* and epigenetic factor genes; SPL, mutations in splicing factor genes other than *SF3B1*; WT, wild‐type. *P < 0.05, ***P < 0.001.

From the set of circRNAs that were deregulated in *SF3B1*‐mutated patients, a cluster of several *ZEB1*‐derived circRNAs was chosen for further investigation. This selection was made given to the strong significance of their deregulation and consistency of the changed expression of multiple *ZEB1*‐circRNAs and based on the functioning of *ZEB1* as a transcription factor related to cancer and hematopoiesis [30]. In our RNA sequencing data, the expression of *ZEB1*‐circRNAs was significantly increased in *SF3B1*‐mutated MDS patients in comparison to healthy controls and nonmutated MDS patients. We observed substantial alterations in both linear splicing and backsplicing, although the expression of *ZEB1* remained unchanged at the whole gene level. Interestingly, Fig. 2 suggests that the majority of these changes occurred mainly in the central region of the *ZEB1* gene.

**Fig. 3:**
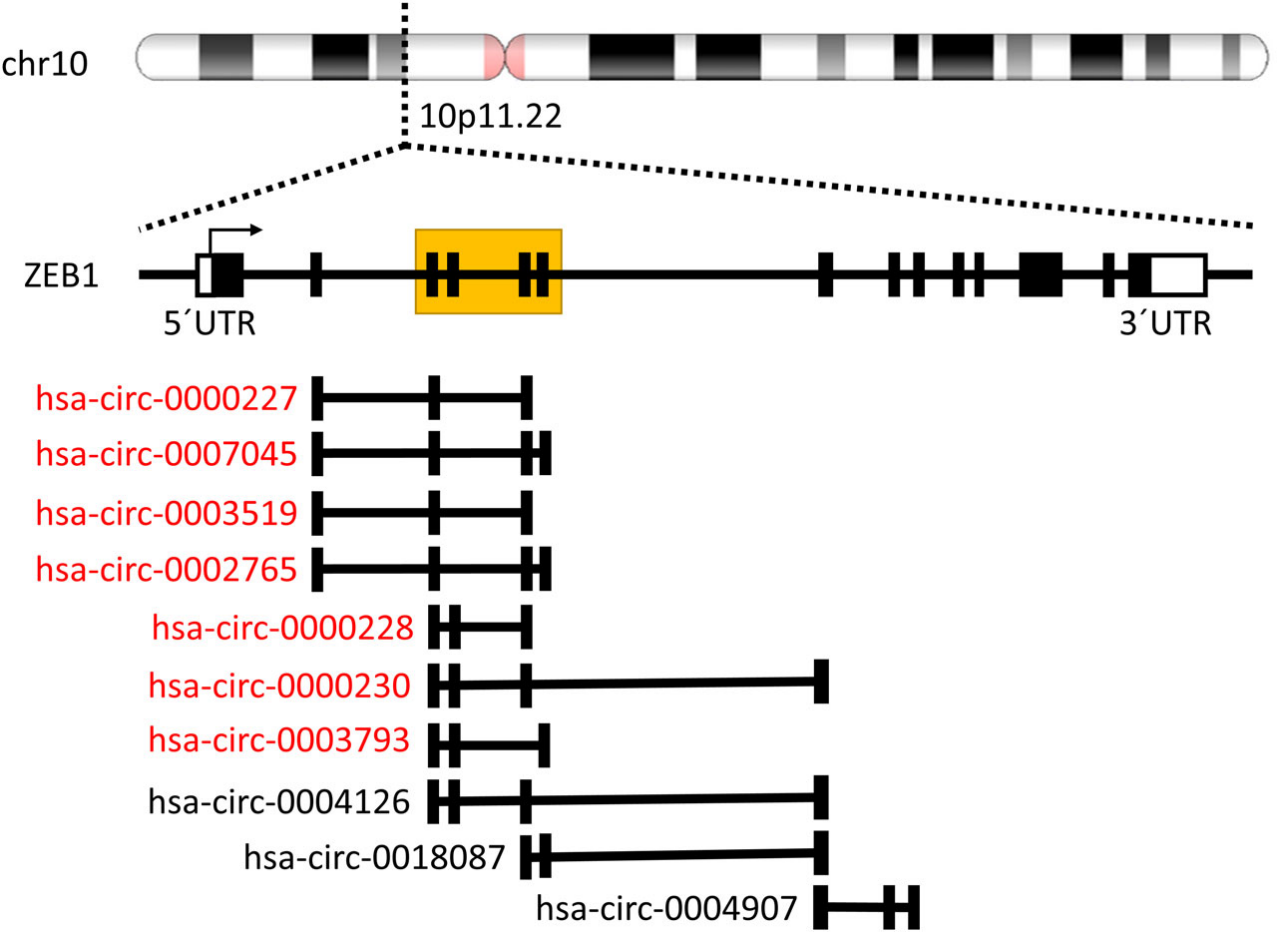
Schema of the *ZEB1* locus. circRNAs with increased expression in *SF3B1*‐mutated patients are highlighted in red. The minimal region seen in the circRNA upregulation is yellow squared.

Multiple transcript variants encoded by *ZEB1* are known (41 RefSeq mRNAs, six antisense mRNAs and 26 circRNAs). We therefore examined the expression of these different forms and their correlations. Of 26 circRNAs annotated in the circInteractome database, we detected 10 circRNAs by RNA sequencing. Of these circRNAs, the expression of seven circRNAs (hsa_circ_0000227, hsa_circ_0000228, hsa_circ_0000230, hsa_circ_0002765, hsa_circ_0003519, hsa_circ_0003793 and hsa_circ_0007045) significantly correlated (*P* < 0.05) with each other, whereas the expression of three circRNAs (hsa_circ_0004126, hsa_circ_0004907 and hsa_circ_0018087) remained independent (Table S5). The scheme of the gene structure presented in Fig. 3 shows that the upregulated circRNAs consist of four alternatively spliced exons, but all of them share the same minimal region located at the central part of the gene, suggesting that this region (chr10:31661947‐31676195; hg19) is essential for the upregulation of circRNA expression. Interestingly, the expression of two linear transcripts (ENST00000488625 and ENST00000559496) also correlated with circRNA levels, and both of them retained the same exons as the circRNAs.

Comparison of the number of BSJ reads in RNA sequencing data showed that hsa_circ_0000228 is the most highly expressed *ZEB1*‐circRNA in *SF3B1*‐mutated patients. It has more than 5‐fold higher expression than the other upregulated *ZEB1*‐circRNAs (Table S5). Thus, we particularly focused on hsa_circ_0000228 in subsequent experiments.

### 3.4 Confirmation of the circular nature of upregulated *ZEB1* transcripts

We performed several experiments to prove that the upregulated *ZEB1* transcripts are truly circular in nature. Initially, we treated RNA isolated from *SF3B1*mut NALM6 cells with RNase R exonuclease, which specifically cleaves linear RNA but leaves circRNA intact. Figure S2A shows that although the levels of several linear mRNAs (*HPRT1*, *GAPDH*, *SF3B1* and *ZEB1*) were significantly reduced after treatment, the levels of seven circRNAs backspliced from *ZEB1* remained almost unchanged. Furthermore, we performed Sanger sequencing to confirm the backsplicing site of hsa_circ_0000228 and hsa_circ_0003793 (Fig. S2B). Finally, actinomycin D treatment of *SF3B1*mut NALM6 cells was used to block RNA transcription for comparison of the stability of circular and linear transcripts. The results showed hsa_circ_0000228 is more stable that linear *ZEB1* mRNA (Fig. S2C).

### 3.5 Upregulation of *ZEB1*‐circRNAs is specific for *SF3B1*‐mutated MDS

We examined the expression of *ZEB1*‐circRNAs with respect to various clinical variables. We proved that deregulation of the backsplicing process is specifically associated with the presence of *SF3B1* mutation and no other tested clinical characteristics. Age, sex, MDS subtype, prognostic IPSS‐R category, karyotype and blast count did not correlate with ZEB1‐circRNA levels. In patient samples, level of ZEB1‐circRNAs significantly correlated with VAF of SF3B1 mutation (hsa_circ_0000228: Pearson *r* = 0.689, *P* < 0.001). Importantly, *ZEB1*‐circRNA upregulation was specific exclusively for *SF3B1* mutations and not for mutations in other splicing factors (*SRSF2*, *U2AF1* and *ZRSR2*) or other recurrently mutated genes. As an example, Fig. S3 shows relationships between hsa_circ_0000228 expression and the abovementioned clinical variables.

### 3.6 *ZEB1*‐circRNAs are upregulated as a result of *SF3B1* mutation but not its inhibition

**Fig. 4:**
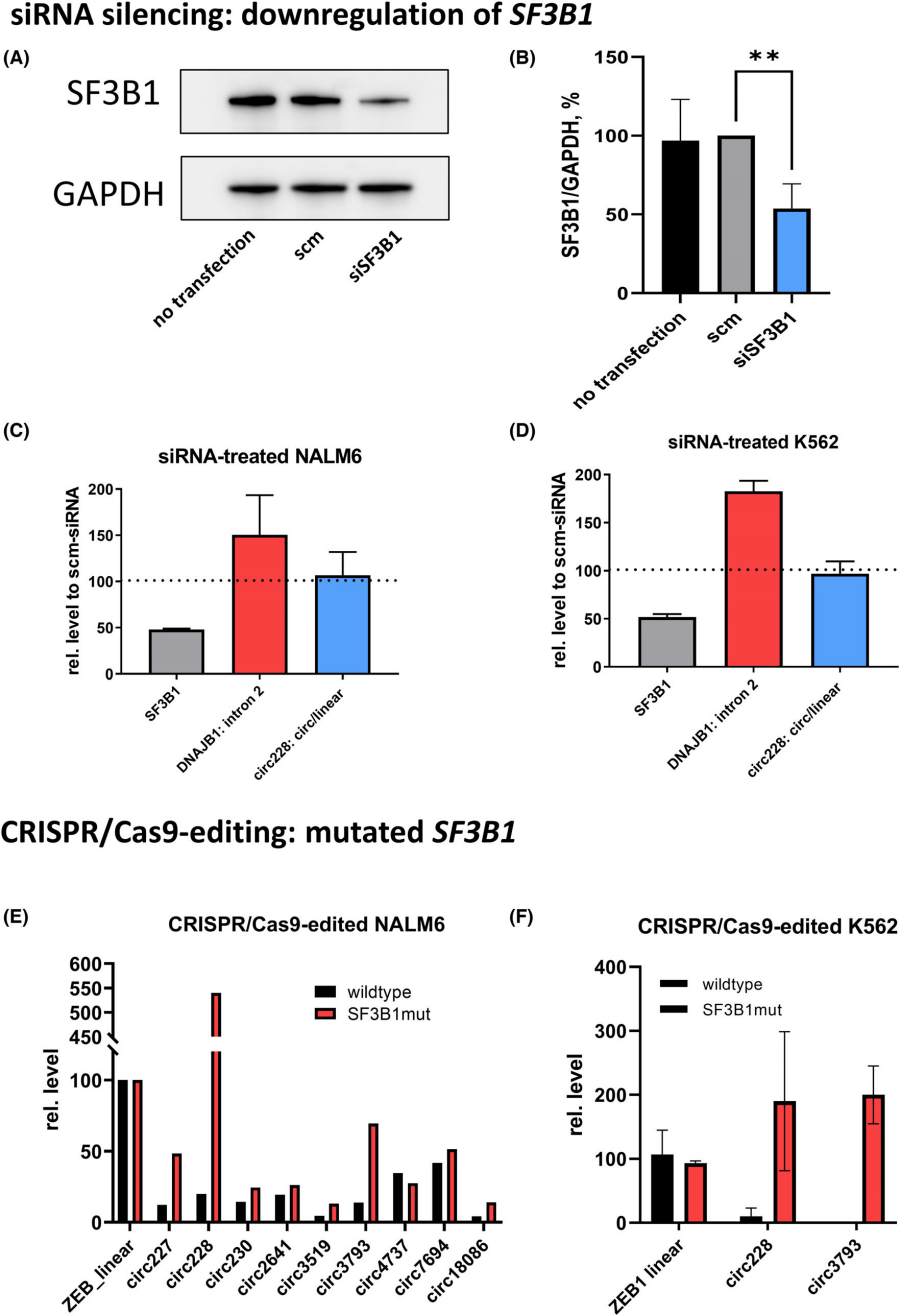
Relationship between *SF3B1* alterations and the expression of *ZEB1*‐circRNAs. (A–D) Effect of siRNA knockdown of *SF3B1* expression. (A) Western blot analysis of SF3B1 protein level after its siRNA silencing in K562 cell line. (B) Quantification of SF3B1 protein level based on western blot image analysis. Student's *t*‐test was used to compare expression values from three different blots. **P < 0.01. (C, D) siRNA knockdown of *SF3B1* expression in *SF3B1*wt NALM6 (C) and K562 (D) cell lines. *SF3B1* transcript level, level of retained intron 2 in *DNAJB1* mRNA and circular‐to‐linear ratio of hsa_circ_0000228 were measured by RT‐qPCR 48 h after *SF3B1*‐siRNA transfection and related to values measured in cells transfected by scm‐siRNA. Data were obtained from three independent experiments and are shown as the mean with SD. (E, F) Expression changes in cell lines with CRISPR/Cas9‐edited *SF3B1* ^K700E^ mutation. (E) Expression of *ZEB1*‐circRNAs in isogenic *SF3B1*wt and *SF3B1*mut NALM6 cell lines. circRNA levels were measured in duplicates by RT‐qPCR and related to the expression of linear *ZEB1*. (F) Expression of *ZEB1*‐circRNAs in isogenic *SF3B1*wt and *SF3B1*mut K562 cells that we originally analysed based on RNA sequencing data downloaded from the publication of Liberante et al. [33]. Data were calculated from two independent experiments and are shown as the mean ± SD. scm, scrambled siRNA; siSF3B1, siRNA targeting *SF3B1*.

To further investigate the association between *SF3B1* mutation and *ZEB1*‐circRNA upregulation, we silenced *SF3B1* expression by an siRNA approach. Using siRNA transfection, we knocked down the level of *SF3B1* to approximately 50% compared to scm‐siRNA in *SF3B1*wt NALM6 and K562 cell lines (Fig. 4A–D). We also analysed *DNAJB1* expression as a surrogate marker for RNA splicing modulation [31] and confirmed that transfection of *SF3B1*‐siRNA increased an unspliced form of *DNAJB1* retaining intron 2 in both cell lines. Nevertheless, the expression of hsa_circ_0000228 did not significantly change after *SF3B1* knockdown (Fig. 4C,D), which can be explained by mutated *SF3B1* in MDS being of change‐of‐function rather than loss‐of‐function nature [32]. Based on this assumption, we hypothesised that the increased circularisation of *ZEB1* transcripts is specifically caused by altered function of SF3B1 and not by its reduced expression.

To test our hypothesis, we examined the consequence of *SF3B1* mutation on hsa_circ_0000228 expression in *SF3B1*wt and *SF3B1*mut NALM6 cell lines. In *SF3B1*mut NALM6, we observed a strong increase in the expression of the majority of *ZEB1*‐circRNAs compared to the isogenic *SF3B1*wt cell line. Interestingly, the level of hsa_circ_0000228 in *SF3B1*mut cells was even 5.3‐fold higher than the level of the linear *ZEB1* form (Fig. 4E). Similar upregulation was detected in CRISPR/Cas9‐edited K562 cells with the *SF3B1* ^K700E^ mutation (we downloaded RNA‐seq data from a publication by Liberante et al. [33] and reanalysed them specifically for expression of circRNAs, which was not considered in the original paper; Fig. 4F). Taken together, these data indicate that the altered function of *SF3B1* caused by the K700E mutation, but not a reduction in *SF3B1* expression, specifically affects splicing in the *ZEB1* locus favouring transcript circularisation.

### 3.7 Hsa_circ_0000228 knockdown results in changes in cellular metabolism

Because the most upregulated circRNA in *SF3B1*‐mutated patient cells as well as in *SF3B1*‐mutated NALM6 cells was hsa_circ_0000228, we specifically focused on this circRNA in subsequent experiments. Based on high sequence overlap, similar functions can be expected for the majority of *ZEB1*‐circRNAs, which are produced from the same locus.

To analyse the functional effects of hsa_circ_0000228, we knocked down hsa_circ_0000228 using siRNA in the *SF3B1*mut NALM6 cell line. Based on RT‐qPCR measurements, we reduced hsa_circ_0000228 levels by 5‐fold compared to the scm‐siRNA negative control, whereas the expression of linear *ZEB1* mRNA and its protein product remained stable (Fig. S4). Initially, we analysed the effects of the knockdown on expression by RNA sequencing. DEA resulted in 33 upregulated (e.g., *ATP11B*, *EPB41L2*, *RSL1D1*, *L3MBTL3* and *UBE2V1*) and 42 downregulated (e.g., *SET*, *SPG11*, *ARHGAP4*, *ACTG1* and *PHTF2*) genes (FDR <0.05). GSEA associated the deregulated genes particularly with RNA processing, such as RNA splicing and degradation (e.g., multiple *LSM* genes – core components of the spliceosomal U6 small nuclear ribonucleoprotein complex, and *U2AF1*), and mitochondrial functions, such as mitochondrial biogenesis and fusion, complex I assembly, electron transport and OXPHOS (particularly multiple *NDUF* genes) and mitochondrial DNA‐encoded genes (particularly many mitochondrial tRNAs) (Fig. S5).

**Fig. 5:**
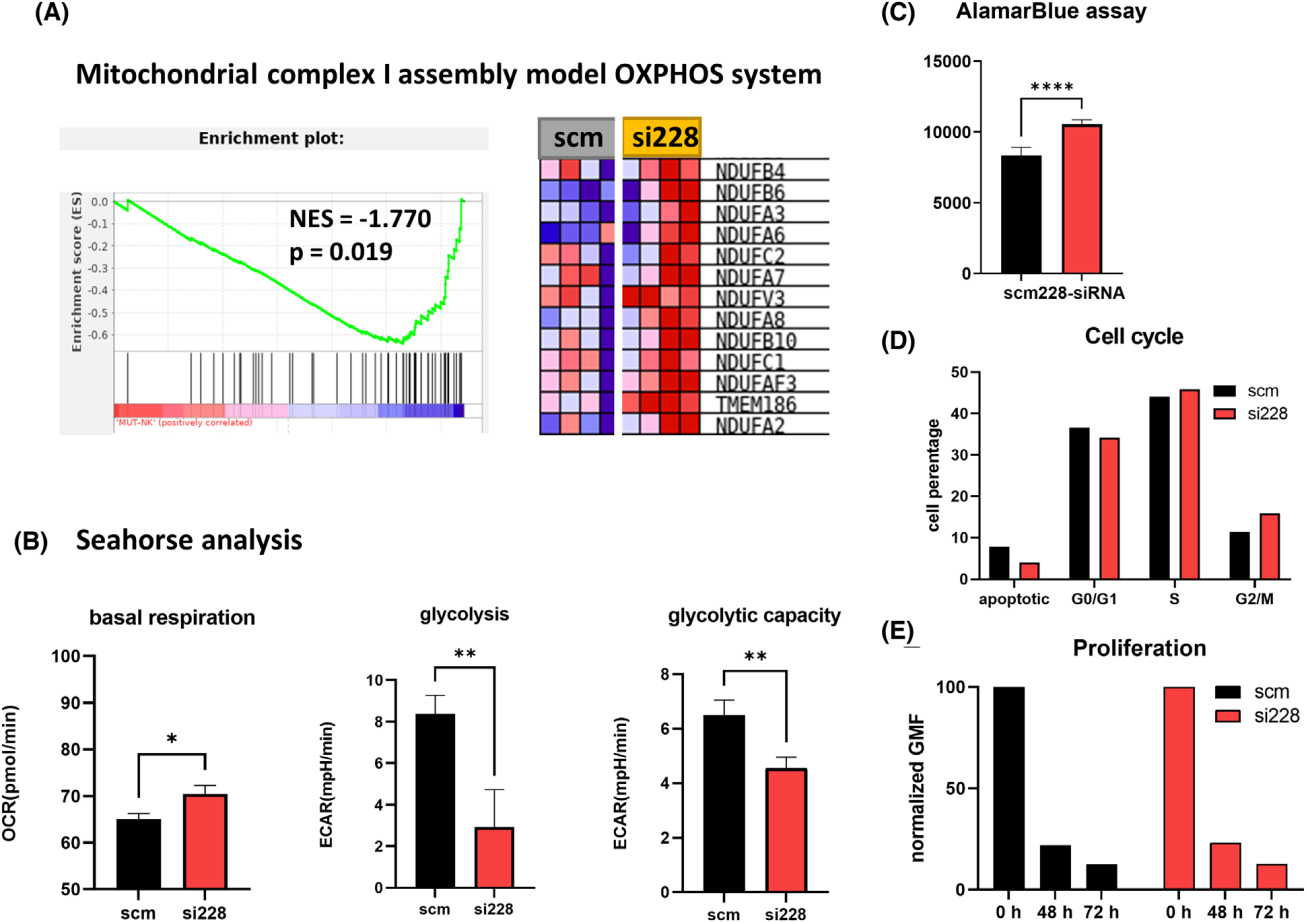
Functional changes in *SF3B1*mut NALM6 48 h after siRNA knockdown of hsa_circ_0000228. (A) GSEA plot of the ‘Mitochondrial complex I assembly model OXPHOS system’ pathway. (B) Seahorse analysis of real‐time OCR and extracellular acidification rate (ECAR). (C) Metabolic activity measured using the AlamarBlue assay. (D) Flow cytometry analysis of the cell cycle. (E) Proliferation measured by dilution of CellTrace Violet™ dye (a lower GMF corresponds to a higher cell proliferation). All data are shown as the mean with SD from at least triplicates. Student's *t*‐test was used to compare expression values, and differences were considered statistically significant at *P* < 0.05. GMF, geometric mean fluorescence intensity; NES, normalised enrichment score; scm, scrambled siRNA; si228, siRNA targeting hsa_circ_0000228; *P < 0.05, **P < 0.01, ****P < 0.0001.

Given that *SF3B1* mutation has been associated with mitochondrial alterations and metabolic reprogramming [34, 35], we further focused on changes in OXPHOS‐related genes and pathways, such as deregulation of complex I gene expression and the assembly model (Fig. 5A). Seahorse metabolic analysis showed that hsa_circ_0000228 knockdown in *SF3B1*mut NALM‐6 cells led to a significant increase in the oxygen consumption rate (OCR) and a decrease in glycolysis (Fig. 5B). These results suggest that hsa_circ_0000228 knockdown may, through induction of mitochondrial complex I, lead to induction of oxidative phosphorylation and reduction of glycolysis. The AlamarBlue assay, which measures the reducing power of cells, demonstrated that NALM‐6 cells after hsa_circ_0000228 knockdown reached higher metabolic activity (Fig. 5C). Flow cytometry monitoring of the cell cycle phases and apoptosis showed a slightly lower percentage of apoptotic cells and a higher rate of G2/M‐phase cells in *SF3B1*mut NALM‐6 cells after hsa_circ_0000228 knockdown (Fig. 5D). However, Trypan Blue exclusion assay and CellTrace™ Violet dye dilution assay (Fig. 5E) did not detect any difference in cell proliferation after hsa_circ_0000228 knockdown, suggesting that metabolic alterations associated with the level of hsa_circ_0000228 are not reflected in changes of cell proliferation.

### 3.8 Hsa_circ_0000228 directly affects miR‐1248 expression

**Fig. 6:**
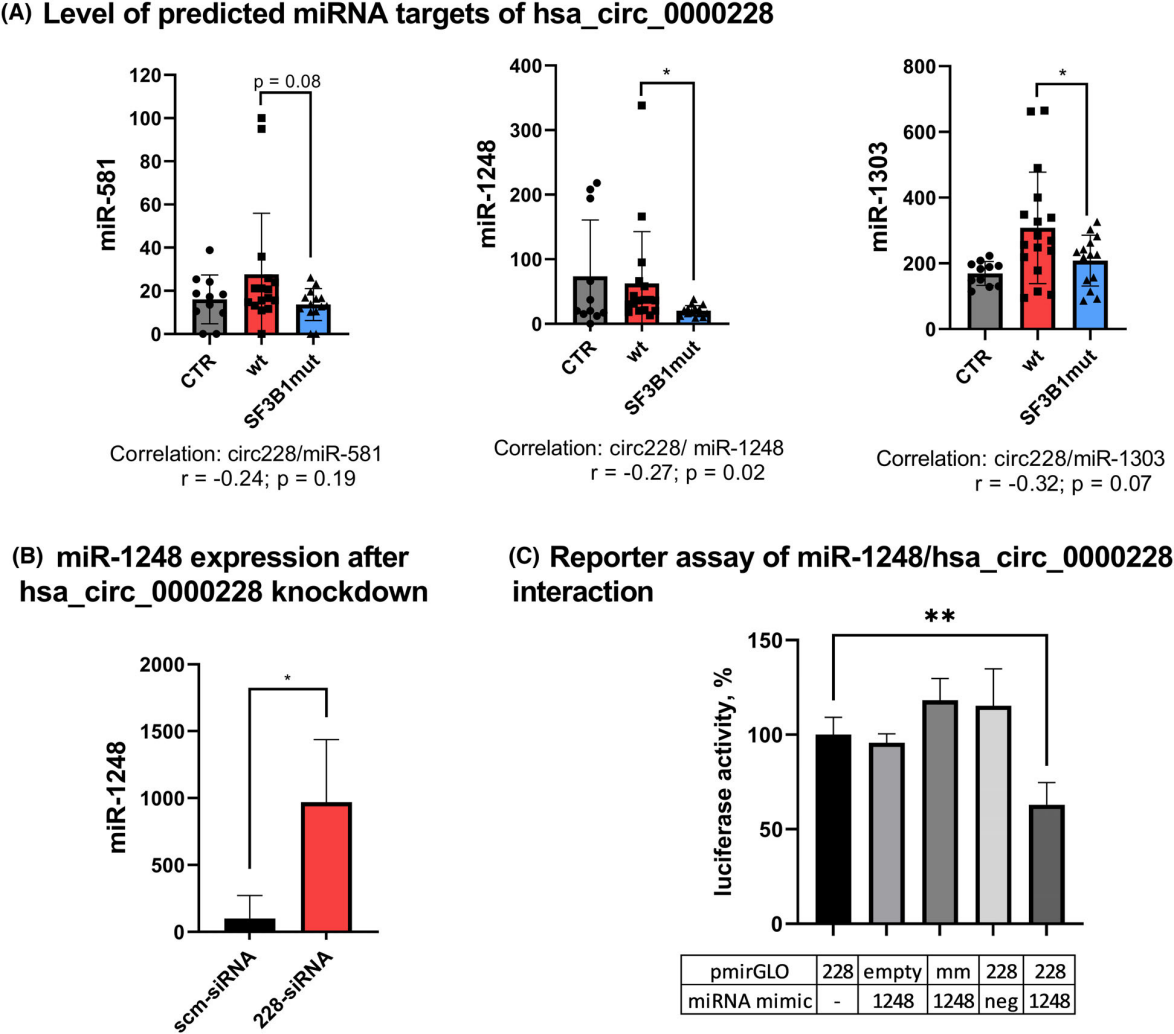
Expression of predicted hsa_circ_0000228 targets: miR‐581, miR‐1248 and miR‐1303. (A) Relative miRNA expression levels in healthy controls (ctr, *n* = 11), MDS patients without any mutation (wt, *n* = 17) and *SF3B1*‐mutated MDS patients (SF3B1mut, *n* = 15) measured by RT‐qPCR and shown as the mean ± SD. (B) Expression of miR‐1248 after siRNA knockdown of hsa_circ_0000228 in *SF3B1*‐mutated NALM‐6 cell line. Data are shown as the mean with SD from triplicates. (C) Luciferase reporter assay proving direct interaction between hsa_circ_0000228 and miR‐1248. K562 cells were cotransfected with pmirGLO vector containing a sequence from hsa_circ_0000228 encoding putative miR‐1248 target site (228), a mismatch sequence of this region (mm), or an empty vector (empty), together with either mimic miR‐1248 or control miRNA (neg). Firefly luciferase activity was measured in triplicates and normalised to constitutive activity of renilla luciferase (shown as the mean with SD). Student's *t*‐test was used to compare expression values. *P < 0.05, **P < 0.01.

CircRNAs most frequently function as inhibitors of miRNAs via binding miRNAs based on their sequence complementarity (so‐called sponging). Using CircInteractome database [36], we identified 11 miRNAs with context score percentile > 90 and as putative targets of hsa_circ_0000228. Further, we combined these data with an output from a novel algorithm circGPA, which we proposed in our preceding study [37]. Based on these two tools, we defined putative miRNA targets for hsa_circ_0000228 (miR‐346, miR‐581, miR‐1184, miR‐865‐5p, miR‐1248 and miR‐1303). Here, we analysed their expression and showed that three of these miRNAs (miR‐581, miR‐1248 and miR‐1303) were downregulated in *SF3B1*‐mutated MDS patients and two of them (miR‐1248 and miR‐1303) showed also a moderate trend of correlation between miRNA and circRNA levels (Fig. 6A). To test whether hsa_circ_0000228 affects levels of these miRNAs, we knocked down hsa_circ_0000228 using an siRNA approach in *SF3B1*mut NALM‐6 cell line. hsa_circ_0000228 downregulation resulted in significant increase of miR‐1248 level (Fig. 6B), whereas levels of miR‐581 and miR‐1303 remained unaffected, assuming that miR‐1248 is a likely target of hsa_circ_0000228.

To confirm the direct interaction of hsa_circ_0000228 with miR‐1248, we used dual‐luciferase reporter assay. We cotransfected miR‐1248 with a reporter pmirGLO plasmid containing a sequence of hsa_circ_0000228 that was predicted as a miR‐1248 binding site in K562 cells and observed downregulation of luciferase reporter activity. No such effect on luciferase activity was seen when the miRNA was cotransfected with the empty vector or a construct carrying a mismatch binding site, or when the vector with the binding site was cotransfected with mimic negative control miRNA, confirming specificity of hsa_circ_0000228 with miR‐1248 interaction. Hence, these results indicate that hsa_circ_0000228 directly binds miR‐1248, affecting its expression (Fig. 6C).

To define signalling pathways affected by miR‐1248, we downloaded its previously validated targets from MirTarBase and performed pathway enrichment analysis using DAVID bioinformatic tool. Based on the list of 163 target genes, we defined seven KEGG pathways associated with miR‐1248 (Table S6). The most significantly (*P* = 0.004) enriched process in the gene list was mitophagy, selective degradation of mitochondria by autophagy, which often occurs to defective mitochondria following damage or stress. Additionally, several cancer pathways were related to miR‐1248 (particularly ErbB and MAPK signalling) through these target genes: *MYC*, *SP1*, *BRCA1*, *CCND2*, *CDKN1A*, *IL5*, *NOTCH2*, *PRKCB*, etc. Thus, miRNA analyses together with pathway enrichment data showed that increased level of hsa_circ_0000228 might lead, through miR‐1248 binding, to deregulation of many key cellular processes, including proliferation, differentiation, apoptosis and stress response.

## 4 Discussion

Our study describes the expression profiles of circRNAs in a large cohort of patients with different subtypes of MDS. Although our major focus was placed on the deregulation of circRNAs caused by somatic mutations in the splicing factor gene *SF3B1*, the study also enabled comprehensive insights into circRNA expression in MDS in general. Using high‐depth RNA sequencing, we identified specific circRNAs whose expression was deregulated in MDS irrespective of patient classification variables and circRNAs with potential prognostic value. Interestingly, we observed a strong trend of circRNA overexpression in higher‐risk patients, associating induction of the backsplicing process with disease severity. In the future, several of these deregulated circRNAs can be tested as new valuable biomarkers of disease progression and/or survival. Recently, a study by Wu et al. [13] proposed six prognosis‐related circRNAs in MDS; however, none of these circRNAs was associated with disease progression based on our data. Large heterogeneity between the two studies can be expected due to different approaches and numbers of subjects/patients in discovery cohorts, as Wu et al. performed profiling analyses using a circRNA array in unsorted BM cells on the cohort of only five MDS samples and four controls.

The hypothesis that triggered this study was that mutations in the splicing factor gene *SF3B1* affect the process of circRNA formation. Based on previous observations made in *Drosophila*, splicing factor depletion shifts the transcription process from canonical mRNA splicing towards increased formation of circRNAs [38]. However, there is still no direct evidence that spliceosome mutations influence the progression of leukaemia by affecting the production of circRNAs; thus, this direction became the major focus of our study. Nevertheless, we observed expression changes in specific circRNA molecules instead of a massive deregulation of the whole circRNA profile. Notably, circRNA expression in several patients with mutations in splicing factors was also investigated as a part of a large AML study [39], and similar to our findings, the mutations also did not reduce or enhance circRNA biogenesis in general. Thus, our attention was particularly paid to *ZEB1*‐circRNAs, which became promising candidates for further detailed characterisation because they were exclusively upregulated in all *SF3B1*‐mutated MDS samples in our cohort, whereas low expression was detectable in the rest of our MDS patients as well as in healthy controls.

Within the RNA sequencing data, we examined the abundances of circRNA molecules and compared these abundances to the expression of corresponding linear transcripts. The data showed that changes in the expression of circRNAs are often specific and do not mirror whole gene expression, supporting the theory that the backsplicing process is specifically regulated and may serve special purposes. In the case of the gene of interest, *ZEB1*, the expression was stable in the entire MDS cohort, but circularisation strongly increased specifically in *SF3B1*‐mutated MDS. The *ZEB1* gene encodes a zinc finger transcription factor that represses T‐lymphocyte‐specific *IL2* expression. It plays a pivotal role in solid cancer metastasis by allowing cancer cells to invade and disseminate due to the transcriptional regulation of epithelial‐to‐mesenchymal transition (EMT) [40]. *ZEB1* recruits histone deacetylases, DNA methyltransferase (DNMT) and ubiquitin ligase to the promoter of E‐cadherin, promoting its epigenetic repression and affecting EMT [40]. Recent studies have demonstrated that *ZEB1* is also an essential hematopoietic transcription factor governing blood lineage commitment and fidelity [30]. For example, Almotiri et al. [41] showed that loss of *ZEB1* in HSCs caused self‐renewal defect and multilineage differentiation block, leading to enhanced cell survival, diminished mitochondrial metabolism, ribosome biogenesis, and differentiation capacity, and activating transcriptomic signature associated with AML signalling. Furthermore, *ZEB1* loss in leukaemic stem cells accelerated AML progression, implicating *ZEB1* as a tumour suppressor [41]. Here, we focused particularly on circRNAs processed form *ZEB1* gene. Expression levels of linear and circRNAs originating from the *ZEB1* gene were independent; thus, we investigated the specific roles of *ZEB1*‐circRNAs in this study.

By *in vitro* functional studies using siRNA knockdown, we showed that hsa_circ_0000228, the most upregulated *ZEB1*‐circRNA, may be involved in mitochondrial functions of MDS cells. Brian Dalton et al. [35] reported that *SF3B1* mutations decreased cellular respiration and reduced citric acid cycle metabolites. These changes were accompanied by decrease in mitochondrial complex III of the electron transport chain mediated through missplicing and downregulation of the complex III assembly factor, *UQCC1*. Furthermore, Hsu et al. [34] differentiated MDS‐induced pluripotent stem cells, recapitulating a progressive decrease in hematopoietic differentiation potential. They demonstrated that *SF3B1* perturbed mitochondrial function, leading to the accumulation of damaged mitochondria during disease progression, resulting in apoptosis and ineffective erythropoiesis. However, detailed identification of direct targets of *SF3B1* that contribute to mitochondrial dysfunction and ring sideroblast formation remained elusive. Our data showed that circRNAs, at least those processed from *ZEB1* gene, can affect cellular processes in MDS and may also contribute to mitochondrial dysfunction in *SF3B1*‐mutated MDS.

In literature, the function of several *ZEB1*‐circRNAs, including hsa_circ_0000228, has been described in several types of cancer, such as cervical cancer [42, 43], breast cancer [44] and hepatocellular carcinoma [45, 46]. These studies reported that *ZEB1*‐circRNAs serve as oncogenic factors, enhancing cell proliferation, migration, and invasion, and thus promoting cancer progression. In agreement with our results, all the studies showed that *ZEB1*‐circRNAs are upregulated in cancer; however, they identified different targets and functional axes (miR‐337‐3p/*TGFBR1* [42], mR‐195‐5p/*LOXL2* [43], miR‐448/*EEF2K* [44], miR‐200a‐3p/CDK6 [45] and miR‐199a‐3p/*PIK3CA* [46]).

The lack of overlap between the results of these studies suggests that the axes by which these circRNAs regulate cell behaviour are highly specific for individual cancer types; thus, their function may also be unique in *SF3B1*‐mutated MDS. Indeed, here we showed that levels of hsa_circ_0000228 negatively correlates with miR‐1248 expression in *SF3B1*‐mutated cells, and that hsa_circ_0000228 directly interacts with miR‐1248. Based on pathway analysis, we further proposed that hsa_circ_0000228 may lead, through miR‐1248 sponging, to deregulation of mitophagy (i.e., degradation of defective mitochondria) or several cancer‐related genes and pathways. It is noteworthy, that impaired mitophagy was previously found in erythroid precursors in MDS patients, which might be associated with early apoptosis and ineffective erythropoiesis [47]. Important role for miR‐1248 has repeatedly been described in several types of tumours, affecting cancer cell viability, proliferation, invasion and migration [48, 49, 50, 51, 52]. Although precise role of miR‐1248 in *SF3B1*‐mutated MDS still remains to be elucidated, our data suggest that regulatory axis *SF3B1*mut/ZEB1‐circRNAs/miR‐1248 may at least partly contribute to specific phenotype of this subtype of MDS.

## 5 Conclusions

In conclusion, our findings demonstrate circRNA expression profiles in a large cohort of MDS patients from different disease categories and underline increased circRNA formation in higher‐risk MDS. In MDS patients with mutations in the splicing factor gene *SF3B1*, we did not detect changes in the global production of circRNAs. Instead, we showed a strong increase in the circularisation of *ZEB1* transcripts. Finally, we demonstrated that depletion of hsa_circ_0000228, the most upregulated *ZEB1*‐circRNA in *SF3B1*‐mutated MDS, affects mitochondrial functions and interacts with cancer‐related miR‐1248. In summary, the data presented in our study show that circRNA profiling can improve our knowledge of myelodysplasia by including another, not much recognised, layer to complex regulatory machinery influencing pathophysiological processes in hematopoietic cells.

## Conflict of interest

The authors declare no conflict of interest.

## Author contributions

MDM was involved in conceptualisation, funding acquisition and writing of manuscript; IT and AHr were involved in investigation, data interpretation and writing of manuscript; ZK and KS were involved in RNA sequencing; DK, JK and PR were involved in bioinformatics; AHo was involved in cloning; KS and JD were involved in protein analyses; LJ, SV, JM and JF were involved in cell analyses; MK, JV and MB were involved in mutational screening; JC and AJ were involved in samples and clinical data.

## Supporting information


**Fig. S1.** Venn diagram of differentially expressed genes, linear transcript variants and circRNAs between *SF3B1*‐mutated MDS patients and those with no mutation detected.
**Fig. S2.** Confirmation of the circular nature of upregulated *ZEB1* transcripts.
**Fig. S3.** Relationship between hsa_circ_0000228 expression and major clinical variables.
**Fig. S4.** Effect of siRNA knockdown of hsa_circ_0000228 in isogenic *SF3B1*wt and *SF3B1*mut NALM6 cell lines.
**Fig. S5.** RNA sequencing of *SF3B1*mut NALM6 cells after knockdown of hsa_circ_0000228.
**Table S1.** Characteristics of the cohort.
**Table S2.** Primers and probes designed for *ZEB1*‐circRNAs.
**Table S3.** Significantly deregulated features in differential expression analyses.
**Table S4.** Genes with specific expression changes between *SF3B1*‐mutated MDS patients and those with no mutation detected.
**Table S5.** Genomic and transcriptomic data on *ZEB1*‐circRNAs detected by RNA sequencing.
**Table S6.** KEGG pathways enriched in a set of previously validated targets of miR‐1248.

## Data Availability

Raw data were deposited in the NCBI SRA database (BioProject ID PRJNA896500).
