# Supplementary material for: Expression of circular RNAs in myelodysplastic neoplasms and their association with mutations in the splicing factor gene SF3B1
Source: Mol Oncol. 2023 Jul 17;17(12):2565–83. doi: 10.1002/1878-0261.13486 (PMC10701770; doi:10.1002/1878-0261.13486)

Expression of circular RNAs in myelodysplastic neoplasms and their association with mutations in the splicing factor gene *SF3B1*

Iva Trsova, Andrea Hrustincova, Zdenek Krejcik, David Kundrat, Aleš Holoubek, Karolina Staflova, Lucie Janstova, Sarka Vanikova, Katarina Szikszai, Jiri Klema, Petr Rysavy, Monika Belickova, Monika Kaisrlikova, Jitka Vesela, Jaroslav Cermak, Anna Jonasova, Jiri Dostal, Jan Fric, Jan Musil, and Michaela Dostalova Merkerova

SUPPLEMENTARY INFORMATION

SUPPLEMENTARY TABLES

SI Table 1. Characteristics of the cohort.

**Variable**	
Number of samples	98
Healthy controls	**13**
Gender (male/female)	6/7
Age, mean (range)	41 (27-69)
Patients	**85**
Sex (male/female)	41/44
Age, mean (range)	64 (27-83)
Diagnosis<br>(SLD/MLD/RS/5q-/EB1/EB2/AML-MRC)	9/23/10/8/9/19/7
IPSS-R category<br>(very low/low/intermediate/high/very high/n.a.*)	20/24/13/8/8/12
Marrow blasts [%]: mean (range)	7.0 (0.0-27.4)
Hemoglobin (g/L): mean (range)	102 (66-149)
Neutrophils (× 10^9/L): mean (range)	2.5 (0.1-14.5)
Platelets (× 10^9/L): mean (range)	192 (16-597)
IPSS-R karyotype<br>(very good/good/intermediate/poor/very poor/n.a.)	3/62/6/2/7/5
Cytogenetic features<br>normal karyotype<br>isolated del(5q)<br>isolated del(20q)<br>isolated +8<br>-Y<br>complex<br>other<br>n.a.	 44<br>12<br>2<br>3<br>3<br>9<br>7<br>5
Somatic mutations<br>no. of mutations per patient: 0/1/2/3/4/5/6<br>splicing factors (*SF3B1*/*SRSF2*/*U2AF1*/*ZRSR2*)<br>epigenetic factors (*DNMT3A*/*TET2*/*ASXL1*)<br>*RUNX1*<br>*TP53*	 18/26/21/13/4/1/2<br>25/6/5/2<br>15/12/8<br>9<br>8

n.a. - not analyzed, SLD – single lineage dysplasia, MLD – multilineage dysplasia, RS – MDS with ring sideroblasts, EB – excess of blasts, AML-MRC - acute myeloid leukemia with myelodysplasia-related changes, IPSS-R - revised international prognostic scoring system. *IPSS-R category cannot be assessed in MDS patients without complete clinical data, and it is also not applicable in AML-MRC cases.

SI Table 2. Primers and probes designed for *ZEB1*-circRNAs.

**Sanger sequencing**		
hsa_circ_0000228	Forward primer	5′-AGGTGTGGGGTGTGAGAACT-3′
	Reverse primer	5′-CAAGCAGACAGTAGCCAAATCAC-3′
hsa_circ_0003793	Forward primer	5′-GGGAAAGCCCCTCAAACCATT-3′
	Reverse primer	5′-GCAGACAGTAGCCAAATCACA-3′
**RT-qPCR**		
hsa_circ_0000227	Forward primer	5′-GGTGTGAGAACTTGATCAGTTTGTG-3′
	Reverse primer	5′-AATGCCTGCTCTGTCTTCCTTAAA-3′
	TaqMan probe	5′-CAGCTGCTTTAGAGAACA-3′
hsa_circ_0000228	Forward primer	5′-GGTGTGAGAACTTGATCAGTTTGTG-3′
	Reverse primer	5′-GGCCAATTGCCAGTTGAGAAT-3′
	TaqMan probe	5′-CCTTGTTCTCTAAAGTTTTC-3′
hsa_circ_0000230	Forward primer	5′-GCAGTGAAAGAGAAGGGAATGCTAA-3′
	Reverse primer	5′-GGCCAATTGCCAGTTGAGAAT-3′
	TaqMan probe	5′-ACTGAAAATGTCATCCTCCC-3′
hsa_circ_0002641	Forward primer	5′-GGTGTGAGAACTTGATCAGTTTGTG-3′
	Reverse primer	5′-CCTTTTGATAGTCACTCCATTAGTCTTTCT-3′
	TaqMan probe	5′-TTGTTCTCTAAAGGAAAGCAC-3′
hsa_circ_0003519	Forward primer	5′-GGTGTGAGAACTTGATCAGTTTGTG-3′
	Reverse primer	5′-CAATGCCTGCTCTGTCTTCCTTA-3′
	TaqMan probe	5′-CACCTTGTTCTCTAAAGCTGTT-3′
hsa_circ_0003793	Forward primer	5′-AAAGTGTGGAAGCAAAGGTCATCTA-3′
	Reverse primer	5′-GTTTTCGGCCAATTGCCAGTT-3′
	TaqMan probe	5′-AAAAGTTTTCAGTTTAAATTCTC-3′
hsa_circ_0018086	Forward primer	5′-TTGTGTCAGCCACCTTGTTCT-3′
	Reverse primer	5′-CAATGCCTGCTCTGTCTTCCTTA-3′
	TaqMan probe	5′-CACCTTGTTCTCTAAAGCTGTT-3′

SI Table 3. Significantly deregulated features in differential expression analyses (FDR < 0.05). Each analysis compared two different groups of samples (A versus B). In brackets, the outputs for raw p < 0.01 are included.

Features		(A) patients<br>(B) healthy controls	(A) higher-risk MDS<br>(B) lower-risk MDS	(A) no mutation<br>(B) *SF3B1* mutation
genes	upregulated in A	1590	1926	16 (201)
	downregulated in A	664	1535	28 (219)
transcripts	upregulated in A	1035	1755	34 (432)
	downregulated in A	985	1306	70 (628)
circRNAs	upregulated in A	0 (172)	65 (234)	3 (24)
	downregulated in A	1 (32)	3 (12)	2 (16)

SI Table 4. Genes with specific expression changes between *SF3B1*-mutated MDS patients and those with no mutation detected (raw $p < 0.05$).

Deregulated feature	Stable feature	Number	Gene names
Whole gene<br>Transcript variant<br>circRNA	-	17	*SMO, AMOTL1, ARHGAP32, COL24A1, ENND3, DNAJC3, FLI1, FNDC3B, KIAA0319L, MRRF, NFIX, PDE3B, SCLT1, TMTC2, TXNDC16, ZBTB44, ZNF91*
Transcript variant<br>circRNA	Whole gene	33	*ABCC4, ATXN7L1, BIRC2, CDK14, CNTRL, DCTD, EIF4ENIF1, FHIT, FKBP3, GRB10, HGSNAT, ITPA, KDM1A, LRBA, MCTP1, MPP6, MSRB3, NCOA3, NFATC3, NSD2, P4HB, PLCG2, POC1B, RBM39, SETD2, SLTM, SPEN, SPPL3, TFAP4, UBAP2, UTP18, ZEB1, ZFAND4*
Whole gene<br>circRNA	Transcript variant	2	*FBXL17, ZBTB46*
circRNA	Whole gene<br>Transcript variant	125	*AC098484.3, AC138409.2, AC139795.1, AC233976.1, AGPS, AKR1E2, ANKRD36BP2, ARID1B, ATM, ATP8B4, BARD1, BBX, BLMH, C11orf74, C3orf58, CBL, CNST, CPSF6, CSNK1G3, CTBP2, CTDP1, CUL3, CXXC4-AS1, DGKD, DMC1, DNAH14, DNAJC5, DOCK5, DTNBP1, EFCAB13, EGLN1, EIF4G3, ELMO1, EPB41L2, ERCC5, ERICH1, ETV6, FAM53B, FAR2, FLT3, GFPT1, GGA3, GINM1, GKAP1, GLG1, GMFG, GTF2F2, GUSBP11, HDGFL3, HIBADH, HNRNPM, IPO9, ITGB3BP, KIAA1958, KMT2A, LAPTM4B, LDLRAD3, LINC00674, LINC02246, LMBR1L, MAP3K5, MAPK6, MARCH6, MASTL, MCM10, MED13L, MRPS5, MUC20-OT1, MVP, NAALADL2, NEMP1, NETO2, ODR4, PALB2, PHACTR1, PHACTR4, PLXNC1, PRIM2, PRKG2, PRRC2C, PTPRA, RAB3IP, RANGAP1, RERE, RHOBTB3, RN7SL141P, RREB1, RYK, SAFB, SDHAF2, SMARCC1, SPAG9, SPTLC1P1, SRFBP1, ST6GAL2, STK3, STK39, STX6, SUGT1P3, SWAP70, TMEM56, TMOD3, TPM4, TPST1, TRMT2B, TRPM7, TSNAX-DISC1, TTC28, TXNL4B, UBXN2A, UGGT2, UHRF1, VAPA, XPO1, ZC3H14, ZC3H18, ZC3H6, ZDHHC21, ZNF124, ZNF257, ZNF292, ZNF782, ZNF800, ZNF841*

SI Table 5. Genomic and transcriptomic data on *ZEB1*-circRNAs detected by RNA sequencing. circRNAs are sorted based on the "location" column, which is reported according to the hg19 genome assembly. The "correlated expression" column indicates whether a given circRNA is a part of the coregulated group of circRNAs (correlation p value < 0.05 with each circRNA from the group). CPM - counts per million.

***ZEB1*-circRNA**	**Location**	**Genomic length**	**Mature sequence length**	**Mean CPM (wildptype)**	**Mean CPM (*SF3B1*mut)**	**DEA fold change (*SF3B1*mut/ wild type)**	**DEA p value**	**Correlated expression**
hsa_circ_0000227	chr10:31,644,072-31,676,195	32,123	32,123	69	386	5.57	0.005	yes
hsa_circ_0007045	chr10:31,644,072-31,676,727	32,655	32,655	42	89	2.14	0.142	yes
hsa_circ_0003519	chr10:31,644,075-31,676,195	32,120	18,332	55	315	5.79	0.006	yes
hsa_circ_0002765	chr10:31,644,075-31,676,727	32,652	32,652	13	50	4.00	0.078	yes
hsa_circ_0000228	chr10:31,661,946-31,676,195	14,249	461	666	3479	5.22	0.003	yes
hsa_circ_0003793	chr10:31,661,946-31,676,727	14,781	378	108	671	6.20	<0.001	yes
hsa_circ_0000230	chr10:31,661,946-31,750,166	88,220	662	253	638	2.52	0.001	yes
hsa_circ_0004126	chr10:31,661,946-31,791,437	129,491	88,445	48	58	0.10	0.184	no
hsa_circ_0018087	chr10:31,676,052-31,750,166	74,114	74,114	10	0	0.03	0.076	no
hsa_circ_0004907	chr10:31,749,965-31,791,437	41,472	426	31	0	1.20	0.734	no

SI Table 6. KEGG pathways enriched in a set of previously validated targets of miR-1248 (downloaded from MirTarBase). Pathway enrichment analysis was performed using DAVID bioinformatic tool and only significantly ($p < 0.05$) enriched pathways are listed.

**KEGG pathway**	**miR-1248 target genes**	**p-value**
Mitophagy	*CITED2, SP1, ATG9A, CSNK2A1, OPTN*	0.0041
ErbB signaling pathway	*ABL2, MYC, CDKN1A, HBEGF, PRKCB*	0.0075
Chemical carcinogenesis - receptor activation	*MYC, UGT2B28, BIRC5, KPNA6, NOTCH2, PRKCB, RPS6KA3*	0.013
MAPK signaling pathway	*ELK4, MYC, TAOK3, CACNG8, MAP3K2, PRKCB, RPS6KA3*	0.018
Pathways in cancer	*MYC, SP1, BIRC5, BDKRB2, CCND2, CDKN1A, IL2RA, IL5, NOTCH2, PRKCB, VHL*	0.022
Platinum drug resistance	*BRCA1, TOP2B, BIRC5, CDKN1A*	0.029
Breast cancer	*BRCA1, MYC, SP1, CDKN1A, NOTCH2*	0.045

SUPPLEMENTARY FIGURES

SI Figure 1. Venn diagram of differentially expressed genes, linear transcript variants, and circRNAs between *SF3B1*-mutated MDS patients and those with no mutation detected (raw $p < 0.05$).

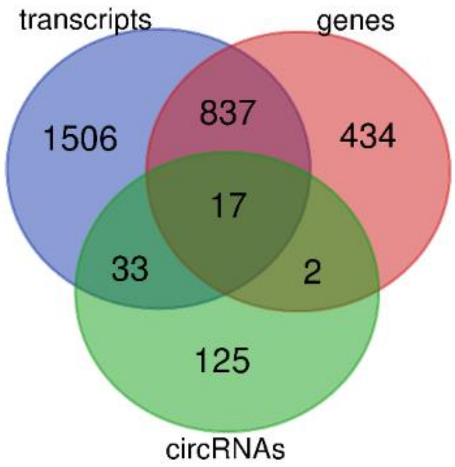

SI Figure 2. Confirmation of the circular nature of upregulated *ZEB1* transcripts. (A) RNase R treatment of RNA isolated from *SF3B1*mut NALM6 cells. RNA was treated with 1 U of RNase R and several linear (*HPRT1, GAPDH, SF3B1,* and *ZEB1*) and circular (seven *ZEB1*-circRNAs) transcripts were quantified by RT-qPCR. Data are visualized as relative expression to those measured in paired samples without RNase R treatment. (B) Sanger sequencing confirming the backsplicing sites of hsa_circ_0000228 and hsa_circ_0003793. (C) Treatment of *SF3B1*mut NALM6 cells with actinomycin D (act; 2.5 µg/mL for 48 hours), an inhibitor of transcription. Cells treated with dimethyl sulfoxide (DMSO) were used as a negative control and quantification was performed by RT-qPCR.

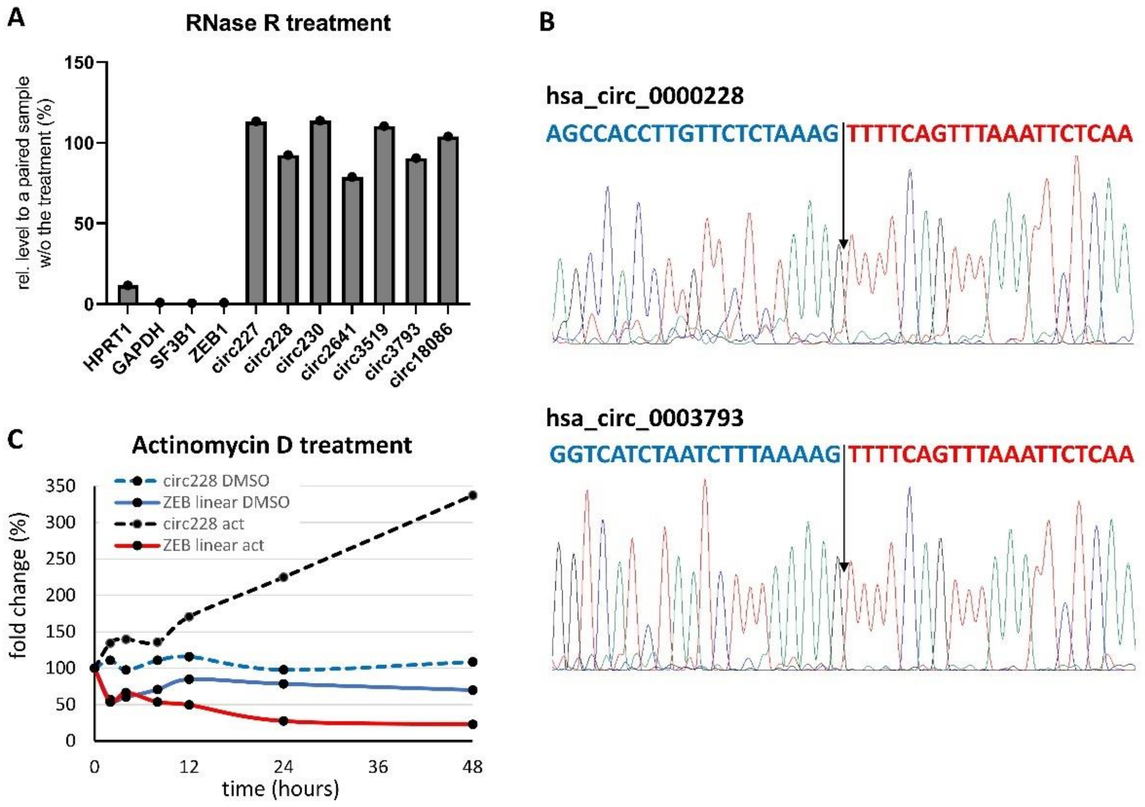

SI Figure 3. Relationship between hsa_circ_0000228 expression and major clinical variables.

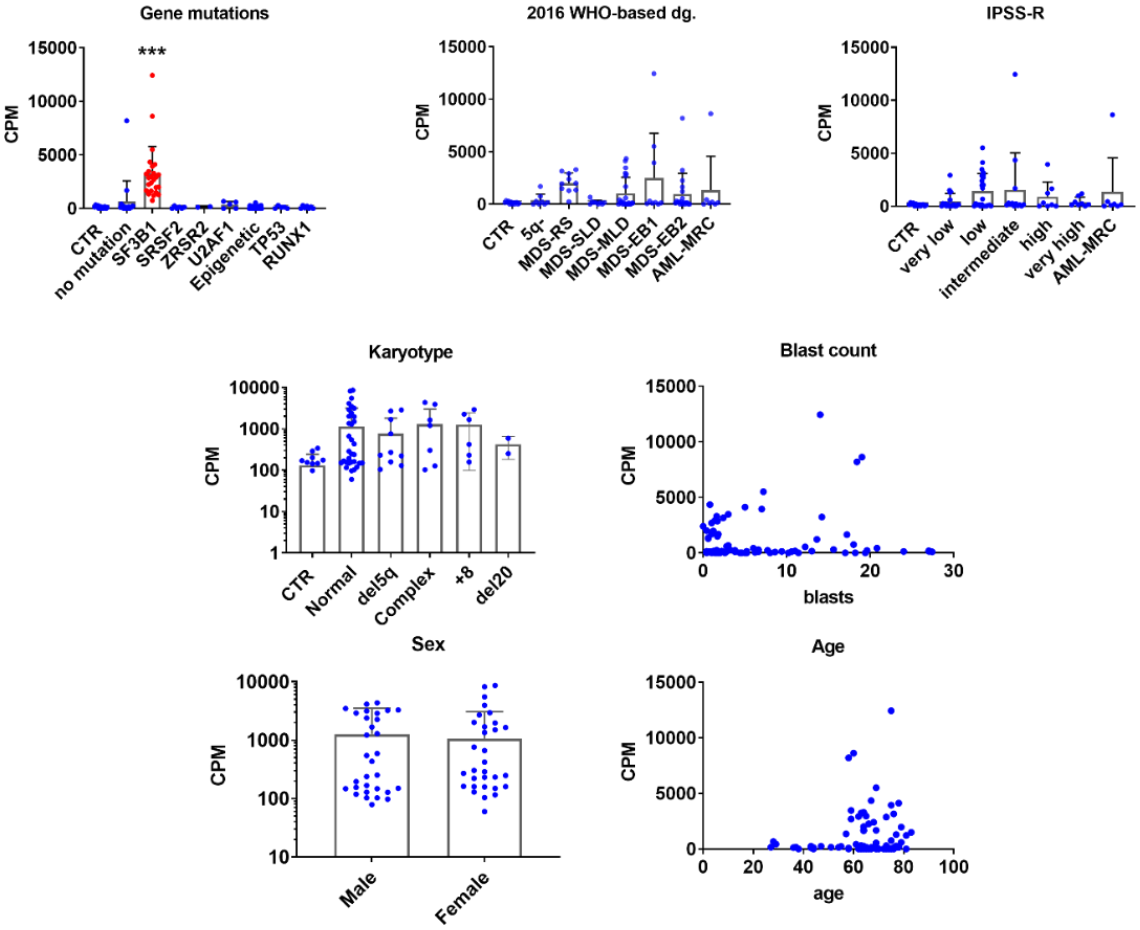

SI Figure 4. Effect of siRNA knockdown of hsa_circ_0000228 in isogenic *SF3B1*wt (WT) and *SF3B1*mut (MUT) NALM6 cell lines monitored 48 h after siRNA transfection by (A) RT-qPCR and (B-C) western blot. The expression of the linear *ZEB1* transcript, ZEB1 protein and hsa_circ_0000228 were related to their expression in *SF3B1*wt NALM6 cells (WT-CTR sample). scm – scrambled siRNA, si228 – siRNA targeting hsa_circ_0000228.

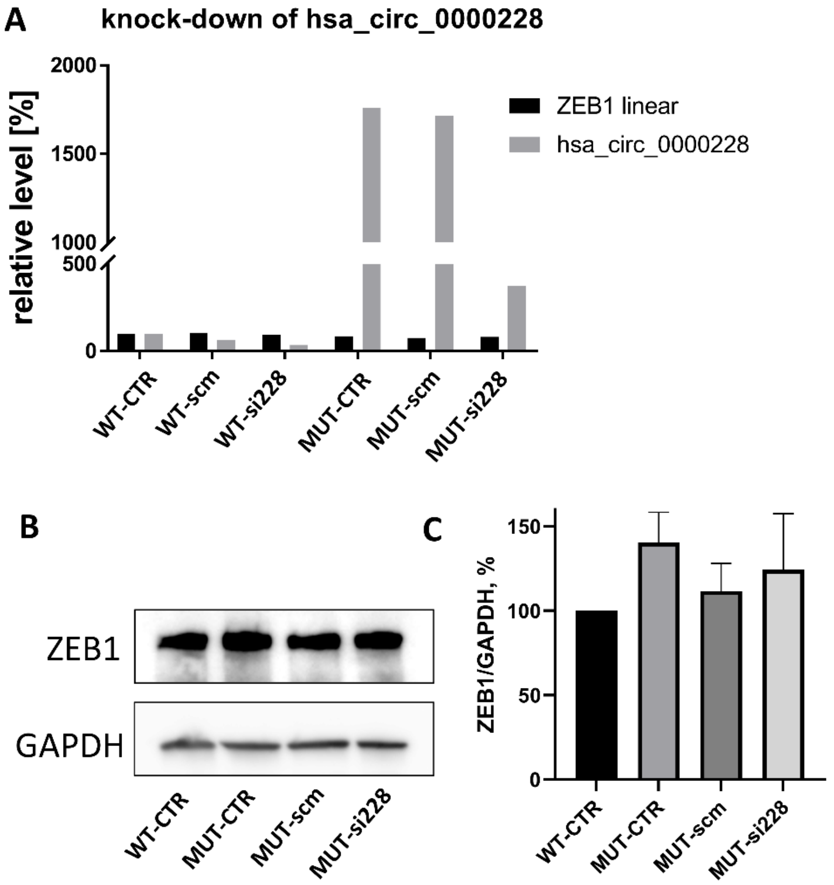

SI Figure 5. RNA sequencing of *SF3B1*mut NALM6 cells after knockdown of hsa_circ_0000228. RNA sequencing was performed 48 h after transfection of siRNA against hsa_circ_0000228. (A) Bar plots show changes in the expression of selected significantly deregulated genes. Expression data are shown as the mean of CPM ± SD calculated from tetraplicates. (B) Alterations in cellular processes and pathways were identified using RNA sequencing results by gene set enrichment analysis (GSEA). scm – scrambled siRNA, NES – normalized enrichment score.

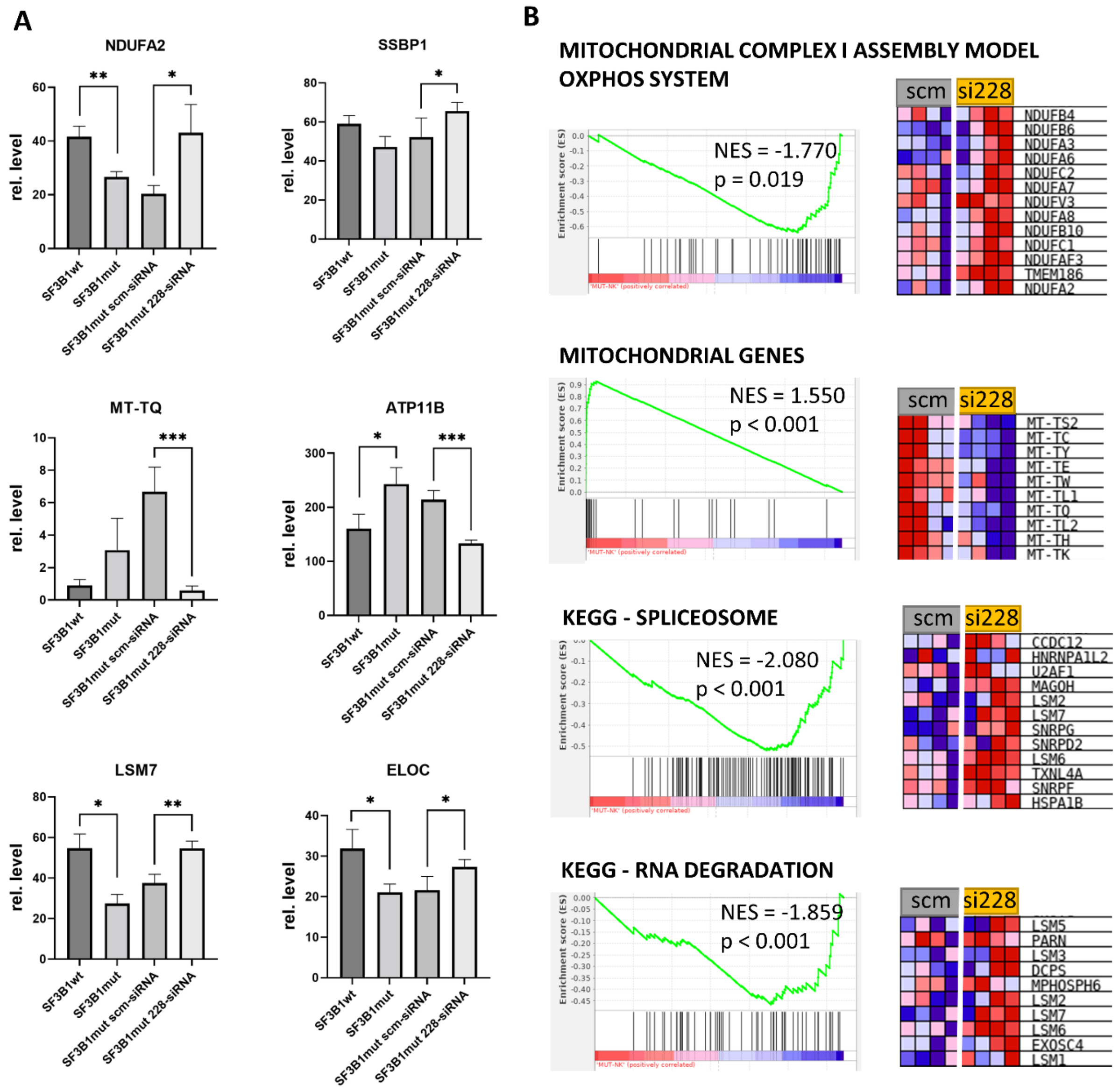

Supplement: Supplementary file 1 — Fig. S1. Venn diagram of differentially expressed genes, linear transcript variants and circRNAs between SF3B1‐mutated MDS patients and those with no mutation detected. Fig. S2. Confirmation of the circular nature of upregulated ZEB1 transcripts. Fig. S3. Relationship between hsa_circ_0000228 expression and major clinical variables. Fig. S4. Effect of siRNA knockdown of hsa_circ_0000228 in isogenic SF3B1wt and SF3B1mut NALM6 cell lines. Fig. S5. RNA sequencing of SF3B1mut NALM6 cells after knockdown of hsa_circ_0000228. Table S1. Characteristics of the cohort. Table S2. Primers and probes designed for ZEB1‐circRNAs. Table S3. Significantly deregulated features in differential expression analyses. Table S4. Genes with specific expression changes between SF3B1‐mutated MDS patients and those with no mutation detected. Table S5. Genomic and transcriptomic data on ZEB1‐circRNAs detected by RNA sequencing. Table S6. KEGG pathways enriched in a set of previously validated targets of miR‐1248. [file MOL2-17-2565-s001.pdf]
